# Role of enterocyte *Enpp2* and autotaxin in regulating lipopolysaccharide levels, systemic inflammation, and atherosclerosis

**DOI:** 10.1016/j.jlr.2023.100370

**Published:** 2023-04-12

**Authors:** Arnab Chattopadhyay, Pallavi Mukherjee, Dawoud Sulaiman, Huan Wang, Victor Girjalva, Nasrin Dorreh, Jonathan P. Jacobs, Samuel Delk, Wouter H. Moolenaar, Mohamad Navab, Srinivasa T. Reddy, Alan M. Fogelman

**Affiliations:** 1 Division of Cardiology, Department of Medicine, Fielding School of Public Health, University of California, Los Angeles, CA, USA; 2 The Vatche and Tamar Manoukian Division of Digestive Diseases, Fielding School of Public Health, University of California, Los Angeles, CA, USA; 3 UCLA Microbiome Center, Fielding School of Public Health, University of California, Los Angeles, CA, USA; 4 David Geffen School of Medicine at UCLA and the Division of Gastroenterology, Hepatology and Parenteral Nutrition, Veterans Administration Greater Los Angeles Healthcare System Los Angeles, Fielding School of Public Health, University of California, Los Angeles, CA, USA; 5 Molecular Toxicology Interdepartmental Degree Program, Fielding School of Public Health, University of California, Los Angeles, CA, USA; 6 Division of Biochemistry, Netherlands Cancer Institute, Amsterdam, the Netherlands; 7 Department of Molecular and Medical Pharmacology, Fielding School of Public Health, University of California, Los Angeles, CA, USA

**Keywords:** lysophospholipase D, lysophosphatidic acid, atherosclerosis, oxidized phospholipids, small intestine, apolipoprotein A-I mimetic peptides, transgenic tomatoes expressing the apoA-I mimetic peptide 6F

## Abstract

Conversion of lysophosphatidylcholine to lysophosphatidic acid (LPA) by autotaxin, a secreted phospholipase D, is a major pathway for producing LPA. We previously reported that feeding *Ldlr*^*−/−*^ mice standard mouse chow supplemented with unsaturated LPA or lysophosphatidylcholine qualitatively mimicked the dyslipidemia and atherosclerosis induced by feeding a Western diet (WD). Here, we report that adding unsaturated LPA to standard mouse chow also increased the content of reactive oxygen species and oxidized phospholipids (OxPLs) in jejunum mucus. To determine the role of intestinal autotaxin, enterocyte-specific *Ldlr*^*−/−*^/*Enpp2* KO (intestinal KO) mice were generated. In control mice, the WD increased enterocyte *Enpp2* expression and raised autotaxin levels. Ex vivo, addition of OxPL to jejunum from *Ldlr*^*−/−*^ mice on a chow diet induced expression of *Enpp2*. In control mice, the WD raised OxPL levels in jejunum mucus and decreased gene expression in enterocytes for a number of peptides and proteins that affect antimicrobial activity. On the WD, the control mice developed elevated levels of lipopolysaccharide in jejunum mucus and plasma, with increased dyslipidemia and increased atherosclerosis. All these changes were reduced in the intestinal KO mice. We conclude that the WD increases the formation of intestinal OxPL, which *i*) induce enterocyte *Enpp2* and autotaxin resulting in higher enterocyte LPA levels; that *ii*) contribute to the formation of reactive oxygen species that help to maintain the high OxPL levels; *iii*) decrease intestinal antimicrobial activity; and *iv*) raise plasma lipopolysaccharide levels that promote systemic inflammation and enhance atherosclerosis.

Lysophosphatidic acid (LPA) has emerged as an important signaling molecule in vascular disease (1, 2). LPA was found to mediate the rapid activation of platelets and endothelial cells induced by mildly oxidized LDL and was present in human atherosclerotic lesions (3). Unsaturated LPA was found to release C-X-C motif chemokine ligand 1 (CXCL1) from endothelial cells, which was subsequently, immobilized on the cell surface to mediate LPA-induced monocyte adhesion, and systemic LPA accelerated the progression of atherosclerosis in mice (4). Blocking the LPA receptors 1 and 3 reduced hyperlipidemia-induced arterial leukocyte arrest and atherosclerosis in the presence of functional CXCL1, indicating that hyperlipidemia-induced monocyte recruitment depends on LPA (4). Mice deficient in LPA receptor 4 had about a 25% reduction in atherosclerotic lesion area in the proximal aorta and arch (5). Genome-wide association studies in humans identified single nucleotide polymorphisms in *PLPP3* (phospholipid phosphatase 3) as a novel locus associated with risk for coronary heart disease that was independent of traditional risk factors (6). The protein product of this gene is lipid phosphate phosphatase 3 (LPP3), which decreases the availability of bioactive lipids including LPA. Reductions in *Plpp3* expression in mice increased plasma LPA levels, increased atherosclerosis, and increased plaque-associated LPA and inflammation (7).

LPA has also been shown to play a role in the clinical events that result from atherosclerosis. After acute myocardial infarction in both mice and humans, there was an increase in autotaxin activity, with increased levels of LPA and inflammatory cells in blood and cardiac tissue (8). Moreover, following acute myocardial infarction in an LPA gain-of-function model, *Plpp3*-specific inducible KO mice, LPA levels were further increased, and there was higher systemic and cardiac inflammation compared with littermate controls (8).

LPA plays an important direct role in not only cardiovascular disease (1, 2, 3, 4, 5, 6, 7, 8) but also gut homeostasis and inflammation. LPA regulates the proliferation and differentiation of intestinal epithelial cells (9). LPA receptor 1 is critical for intestinal epithelial homeostasis and wound closure (10). In addition, LPA receptor 1 is important in maintaining intestinal epithelial barrier function and susceptibility to colitis (11). Mice with global deletion of LPA receptor 1 had decreased expression of tight junction proteins in their intestine, reduced intestine barrier function, and increased bacteria loads in the intestinal mucosa and peripheral organs (11). Moreover, these mice had increased susceptibility to develop colitis (11).

We previously reported that the levels of unsaturated LPA in the small intestine correlated with the extent of aortic atherosclerosis (12), and feeding a Western diet (WD) increased unsaturated LPA levels in the small intestine of *Ldlr*^*−/−*^ mice (13). Adding unsaturated LPA to standard mouse chow to achieve levels of LPA in the small intestine comparable to levels seen on feeding a WD resulted in gene expression in the small intestine that was similar to that found on feeding a WD and was associated with dyslipidemia (13), systemic inflammation (13), and aortic atherosclerosis (14) that was qualitatively similar to that induced by feeding the mice a WD.

A major source of LPA is the conversion of lysophosphatidylcholine (LPC) to LPA by autotaxin (phospholipase D), which is the protein product of the *Enpp2* gene (15). Supplementing standard mouse chow with LPC 18:1 resulted in increases in unsaturated LPC 18:1 in jejunum equal to that seen on feeding *Ldlr*^*−/−*^ mice a WD (16), increased jejunum LPA species (14), and also resulted in dyslipidemia similar to that seen on adding unsaturated LPA to chow. Moreover, adding a specific oral inhibitor (PF8380) of autotaxin to mouse chow together with LPC 18:1 reduced these changes suggesting that a significant portion of LPA was being derived from autotaxin-mediated hydrolysis of LPC 18:1 (14). Pancreatic phospholipase A_2_ group 1B is secreted into the duodenum in response to fat in the diet and acts on dietary phospholipids to convert them to their LPC forms that are rapidly and efficiently taken up by the enterocytes of the small intestine (17).

Approximately two-thirds of the fatty acids in the WD used in our studies are saturated. After absorption, dietary phospholipids containing saturated fatty acids are remodeled in enterocytes via LPC acyltransferase 3 (*Lpcat3*). Feeding *Ldlr*^*−/−*^ mice a WD or feeding them chow supplemented with unsaturated LPA equally induced gene expression for *Lpcat3* (14). The importance of phospholipid remodeling and de novo synthesis of unsaturated LPC in small intestinal enterocytes in the induction of WD-mediated systemic inflammation was demonstrated in mice with intestinal-specific knockout of stearoyl-Co-A desaturase-1 (*Scd1*) (18).

The interface between the lumen of the intestine, which is rich in bacteria, and the enterocytes is largely composed of mucus that is secreted by intestinal goblet cells. In the colon, there are two mucus layers; a very dense layer adjacent to the enterocytes that prevents the luminal bacteria from directly interacting with the enterocytes, and a loose layer on the luminal side of the dense layer. The bacteria in the colon reside in the loose layer (19, 20). In the small intestine where fat absorption occurs, there is no dense inner layer and the loose mucus layer is much thinner; it is even intermittent in some areas. In the absence of the dense mucus layer in the small intestine, the separation of bacteria from enterocytes is dependent on antibacterial peptides and proteins that are secreted into the mucus to regulate the number of bacteria and their interaction with the enterocytes (21).

We recently reported (22) that oxidized phospholipids (OxPLs) in the small intestine cause changes in the mucus layer that result in increased lipopolysaccharide (LPS) levels in small intestine and systemic inflammation (22). The 6F peptide is a member of a family of class A amphipathic helical peptides that bind OxPLs so avidly that they cannot interact with cells (23, 24). When *Ldlr*^*−/−*^ mice were fed a chow diet or a WD or a WD to which a concentrate of transgenic tomatoes expressing the 6F peptide (Tg6F) was added, the mucus levels of OxPLs in jejunum of mice receiving Tg6F did not increase beyond the levels seen on feeding the mice a chow diet (22).

After feeding the WD, gene expression in the jejunum decreased for multiple antimicrobial peptides and proteins that are secreted into the mucus layer of the jejunum (22). As expected with such changes, LPS levels in jejunum mucus increased, and there was an increase in LPS levels in the lymph draining the jejunum, and in the plasma of mice fed a WD compared with mice fed chow (22). Adding Tg6F to the WD reduced these WD-mediated changes (22).

Adding OxPLs ex vivo to the jejunum from mice fed a chow diet reproduced the changes in the expression of genes that control the intestinal levels of antimicrobial peptides and proteins in vivo in response to a WD (22), and adding the 6F peptide to the ex vivo incubations prevented the OxPL-mediated changes (22).

The present study was designed to determine the role of LPA produced locally by autotaxin in enterocytes from *Ldlr*^*−/−*^ mice in WD-induced dyslipidemia, systemic inflammation, and atherosclerosis. The results demonstrate additional similarities to those previously reported (13, 14) for feeding *Ldlr*^*−/−*^ mice fed a WD compared with feeding them a chow diet supplemented with LPA. The present study also shows that enterocyte-specific deletion of the gene for autotaxin, *Enpp2*, produced results similar to those obtained on adding Tg6F to a WD that was fed to *Ldlr*^*−/−*^ mice.

## Materials and methods

### Materials

Supplemental Table S1 shows the sources for ELISA kits. The apoA-I mimetic peptide 6F (12) and a control peptide (22) were described previously. Oxidized 1-palmitoyl-2-arachidonyl-*sn*-glycero-3-phosphocholine was purchased from Avanti Polar Lipids (catalog no.: 870604P). Unless otherwise stated, other materials were from previously cited sources (22).

### Mice and diets

*Enpp2*^*fl/fl*^ mice (25) were crossed with *Ldlr*^*−/−*^ mice that were originally purchased from Jackson Laboratories on a C57BL/6J background and maintained in the breeding colony of the Department of Laboratory and Animal Medicine at the David Geffen School of Medicine at UCLA to generate *Enpp2*^*fl/fl*^/ *Ldlr*^*−/−*^ mice, which are referred to in this article as “Cont.” mice. The *Enpp2*^*fl/fl*^/ *Ldlr*^*−/−*^ mice were crossed with Villin Cre (*VilCre*) mice on a C57BL/6J background that were purchased from Jackson Laboratories (stock #004586) to ultimately yield *Enpp2*^*fl/fl*^/ *Ldlr*^*−/−*^*/VilCre* mice, which are referred to in this article as “iKO” mice (intestinal knockout mice). *Enpp2*^*fl/fl*^/ *Ldlr*^*−/−*^ mice were bred with *Enpp2*^*fl/fl*^/ *Ldlr*^*−/−*^*/VilCre* mice, and the progeny were genotyped to obtain the “Cont.” and “iKO” mice that were used in the experiments. The mice had unlimited access to standard mouse chow (Ralston Purina Rodent Laboratory Chow; catalog no.: 5001; 4.5% fat by weight) prior to the start of experiments. As described previously (22), to ensure that the mice ate all their food, during the experiments, the mice did not have unlimited access to food. During the weeks when the mice were on the experimental diets, the mice were given precisely 4 g of diet per mouse each night (each cage contained four mice; each cage received 16 g of diet each night). When the mice were switched to a WD, it was from Envigo (catalog no.: TD88137; 21% fat by weight). After receiving the diets for 2 weeks or for 5 months, all four groups gained weight (supplemental Fig. S1). Mice receiving the WD had greater weight gain compared with chow, which at 2 weeks (supplemental Fig. S1A) was less in the iKO mice on the WD compared with control mice on the WD, but was not less after 5 months (supplemental Fig. S1B). This experimental design assured that all the mice ate the same amount of food each day, and that all four groups (Cont. mice on chow; Cont. mice on WD; iKO mice on chow; and iKO mice on WD) gained weight over the course of the experiment indicating that the caloric intake even on the chow diet was sufficient. The gender and number of mice in each group is stated in the figure legends or in a supplemental Table. If the age range did not exceed 1 month (e.g., age 2–3 months), only the age range of the mice is stated. If the age range of the mice used in the experiment exceeded 1 month, the mean ± SEM of the group’s age in months is reported. In each experiment, the number of mice of each age was the same for each of the four groups. For example, if there were 16 mice per group with ages ranging between 4 and 7 months, there could be four mice aged 4 months, four mice aged 5 months, four mice aged 6 months, and four mice aged 7 months in each of the four groups. In this example, the age of the mice would be stated as 5.5 ± 0.3 months. The UCLA Animal Research Committee approved all experiments prior to initiation of the study, and the UCLA Division of Laboratory Animal Medicine monitored the mice daily to ensure compliance with all applicable rules and regulations.

## Tissue and cell collection

### Collecting jejunum mucus and isolation of enterocytes from the same segment of jejunum

Jejunum mucus was collected as previously described (22). Enterocytes were prepared as described previously (22, 26), which yielded a purity of enterocytes of ∼83% (26). For determination of autotaxin levels associated with enterocytes, highly purified enterocytes were prepared by flow cytometry. Twelve centimeter sections of the proximal jejunum from each mouse were flushed twice with ice-cold PBS, then inverted, and the ends were ligated with thread. Jejunum sections were then incubated with shaking at 37°C for 15 min in buffer A (1.5 mM KCl, 96 mM NaCl, 8 mM KH_2_PO_4_, 27 mM sodium citrate, 5.6 mM Na_2_HPO_4_, pH 7.3, 0.1 mM PMSF, and 1 mM benzamidine), followed by 30 min shaking at 37°C in buffer B (2.7 mM KCl, 137 mM NaCl, 1.5 mM EDTA, 1.5 mM KH_2_PO_4_, 8.1 mM Na_2_HPO_4_, pH 7.4, 0.5 mM DTT, 0.1 mM PMSF, and 1 mM benzamidine). The tissue was further vortexed gently in buffer B to release the intestinal cells, followed by passing the cell suspension through a 70 μm cell strainer (Corning; catalog no.: 352350). Approximately 15 million cells were then passed through EpCAM enriching microbeads (Miltenyi; catalog no.: 130-105-958) and their corresponding LS columns (Miltenyi; catalog no.: 30-042-401). EpCAM enrichment yielded approximately 5 million cells, which were then washed in fluorescence-activated cell sorting (FACS) buffer (5% FBS in PBS). Cells were pelleted by centrifugation at 4°C and incubated for 45 min on ice with the following antibodies, all of which were used at a 1:100 dilution: EpCAM (BioLegend; catalog no.: 118206), CD45 (BioLegend; catalog no.: 103108), CD44 (BioLegend; catalog no.: 103006), CD31 (Invitrogen; catalog no.: 46-0311-82), F4/80 (Invitrogen; catalog no.: 48-4801-82), and LYVE1 (Invitrogen; catalog no.: 48-0443-82). After the addition of antibodies, the cells were kept under foil to avoid photobleaching. Following antibody staining, the cells were washed twice and then resuspended in FACS buffer before analysis on a FACSAria (BD). Compensation was performed with UltraComp eBeads (Invitrogen; catalog no.: 01-3333-42). To sort enterocytes, gates were constructed relative to unstained control cells to sort for EpCAM^+^ cells, while excluding the following populations: CD45^+^, CD44^+^, CD31^+^, F4/80^+^, and LYVE1^+^.

### Ex vivo studies of jejunum

After an overnight fast, the intestines of *Ldlr*^*−/−*^ mice on a chow diet were gently washed with cold PBS being careful to avoid perforation. The jejunum was isolated and carefully cut in to 2 cm sections. The jejunum sections were cut open and added to tubes containing 1 ml of PBS or containing 1 ml of PBS with the additions described in the figure legends. The tubes were incubated at 37°C for 4 h with gentle mixing on a nutating mixer. The tubes were centrifuged at 13,000 rpm at 4°C for 5 min. The supernatant was carefully aspirated, the tissue was homogenized for 15 s, and immediately frozen. The next day RNA was isolated for RT-quantitative PCR (qPCR) as described below.

### Assays

#### Determination of OxPLs in jejunum mucus

OxPLs were determined in jejunum mucus using an ELISA with the E06 antibody (Avanti Polar Lipids; catalog no.: 330001Sn), a PC-BSA standard (Biosearch Technologies; catalog no.: PC-1011-10), and a 1:10 dilution as described previously (22).

#### Isolation of RNA and gene expression analysis

Total RNA was isolated from mouse enterocytes or from whole jejunum or from jejunum segments using a Qiagen RNA extraction kit (catalog no.: 74106) according to the manufacturer’s instructions. The yield and purity of the isolated RNA was determined using a Nano-Drop spectrophotometer. Samples for real-time qPCR (RT-qPCR) had absorbance 260/absorbance 280 ratios between 1.9 and 2.05. First-strand complementary DNA (cDNA) was synthesized from 1 μg of total RNA using a cDNA synthesis kit (Bio-Rad, catalog NO.: 1708890). The cDNA was diluted 1:10 with nuclease-free water. RT-qPCR was performed on a Bio-Rad CFX96 real-time PCR detection system using SYBR Green Supermix (Bio-Rad; catalog no.: 1708880). Unless otherwise stated, relative abundance of mRNA was calculated by normalizing to GAPDH. Primer sequences are shown in supplemental Table S2.

#### Determination of proteins in jejunum mucus, enterocytes, whole jejunum, and plasma

Total protein concentrations were determined by the Bradford assay (Sigma-Aldrich; catalog no.: B6-916) or by the Lowry method using a Pierce™ Modified Lowry Protein Assay Kit (ThermoFisher; catalog no.: 23240) according to the manufacturer’s instructions.

Unless otherwise stated, the levels of specific proteins were determined by ELISA using kits according to the instructions of the manufacturer as shown in supplemental Table S1. For the determination of autotaxin protein, Western blots were performed and the intensity of the target proteins determined by scans. Briefly, highly purified enterocytes were prepared by flow cytometry as described above. Approximately, 1.5 million highly purified enterocytes from each condition were pelleted by centrifugation. Protein cell lysis buffer (15 μl) and an equal volume of 2× Laemmli sample buffer were added to the cell pellet. The samples were boiled for 5 min at 100°C and loaded on an SDS-PAGE gel 4–20% (Bio-Rad; catalog no.: 4561094). Following electrophoresis, gels were transferred to a PVDF membrane with protein transfer buffer using a semidry transfer apparatus (Bio-Rad). Autotaxin protein was determined by Western blot analysis. Primary antibody against autotaxin was from Santa Cruz Biotechnology, (catalog no.: sc-374222), and the secondary antibody was goat anti-mouse IgG that was obtained from MilliporeSigma (catalog no.: AP124P). The blots were stripped and reprobed with vinculin antibody (Sigma; catalog no.: V4139) and goat anti-mouse secondary antibody that was obtained from MilliporeSigma (catalog no.: AP124P). Protein was quantified by ImageJ analysis of the Western blot. Band intensity was measured in arbitrary units. To normalize for loading variation, the relative levels of autotaxin protein were calculated by dividing the autotaxin protein value by the corresponding value for vinculin protein.

#### Immunohistochemistry

Tissue sections were deparaffinized and antigen retrieval was performed with boiling in citrate buffer (10 mM in PBS, pH 6). Primary antibodies for mucin 2 (MUC2) (Santa Cruz Biotechnology; catalog no.: sc15334), E-cadherin (R&D Systems; catalog no.: AF748), and lysozyme (Invitrogen; catalog no.: PA5-16668) were added (1:200 dilution) to the sections in blocking buffer (10% BSA) and incubated overnight at 4°C. The slides were washed twice with 1× PBS and then stained with secondary antibodies (Invitrogen; catalog no.: A32814 and catalog no.: A10042) and with DAPI (Invitrogen; catalog no.: D1306) for 90 min at room temperature. The slides were washed 1× PBS followed by addition of mounting solution (ThermoFisher; catalog no.: P36931) followed by a coverslip. The negative control omitted the primary antibodies. Images were captured on a Leica DMi8 microscope with either 10× or 20× objective, and LAS X software was used for acquisition. The number of Paneth cells per crypt was determined by counting 10 crypts on each of four different slides taken from three different mice per group so that 120 crypts were counted for each group. The same strategy was used for counting the goblet cells per crypt. To determine the goblet cells per villus, 10 villi were counted on each of six different slides taken from three different mice per group so that 180 villi were counted for each group. The average data for each of the four slides for each group (Paneth cells per crypt and goblet cells per crypt) or each of the six slides for each group (goblet cells per villus) from each of the three mice per group are shown in the figures.

#### Other assays

Plasma was collected, and lipids were determined as previously described (22). LPA was measured by mass spectrometry as described previously (14). LPS in jejunum mucus and plasma was determined using a LAL Chromogenic Endotoxin Quantitation Kit (Thermo Fisher Scientific Pierce; catalog no.: 88282) following the manufacturer’s instructions. Measurement of reactive oxygen species (ROS) in jejunum mucus and plasma levels of IL-6 and serum amyloid A (SAA) were determined as described previously (22).

### Statistical analyses

In comparing more than two groups, ANOVA was performed and followed by multiple comparison tests using version 9.1.2 (GraphPad Software, San Diego, CA). In comparing two groups with an unpaired experimental design, the unpaired two-tailed *t*-test was used and a correction (e.g., Welch’s correction) was applied if the standard deviations were different. Statistical significance was considered achieved if *P* < 0.05. Unless otherwise stated, values are presented as the mean ± SEM.

Almost all the figures and supplemental Figures compare four different groups in each panel with each group shown as a bar graph. Reading figures from left to right in which Cont. mice on chow diet or WD and iKO mice on chow or WD are compared, the first bar graph is Cont. mice on the chow diet (Chow-Cont.). The second bar graph is iKO mice on the chow diet (Chow-iKO). The third bar graph is Cont. mice on the WD (WD-Cont.). The fourth bar graph is iKO mice on the WD (WD-iKO). To facilitate the ability of the reader to see similarities and differences at a glance, Chow-Cont. is colored green. Chow-iKO is colored green unless it is significantly different from Chow-Cont. with the difference being in the same direction as the change in the WD-Cont. (e.g., if both Chow-iKO and WD-Cont. have values lower than Chow-Cont., both will have the same color as the WD-Cont.). WD-Cont. is rose colored unless it is not significantly different from Chow-Cont. in which case it is green. WD-iKO is rose colored unless it is not significantly different from Chow-Cont. or is different from the Chow-Cont. in the opposite direction to the change in the WD-Cont. in which case iKO will be colored green. The asterisks denote significant differences as indicated in the figure legends.

## Results

### Adding unsaturated LPA to standard mouse chow increases ROS, OxPLs, and LPS levels in jejunum mucus

We previously reported (13, 14) that adding unsaturated LPA to normal mouse chow caused changes in *Ldlr*^*−/−*^ mice that qualitatively mimicked feeding the mice a WD (e.g., dyslipidemia, systemic inflammation, and aortic atherosclerosis). More recently, we reported that the WD mediates increases in the levels of ROS, OxPLs, and LPS in jejunum mucus and plasma, which enhance WD-mediated dyslipidemia and systemic inflammation (22). Supplemental Fig. S2 demonstrates that similar to feeding *Ldlr*^*−/−*^ mice a WD, adding LPA 18:1 to standard mouse chow increased the levels of ROS, OxPLs, and LPS in jejunum mucus and plasma.

### *Enpp2* expression in enterocytes

**Fig. 1:**
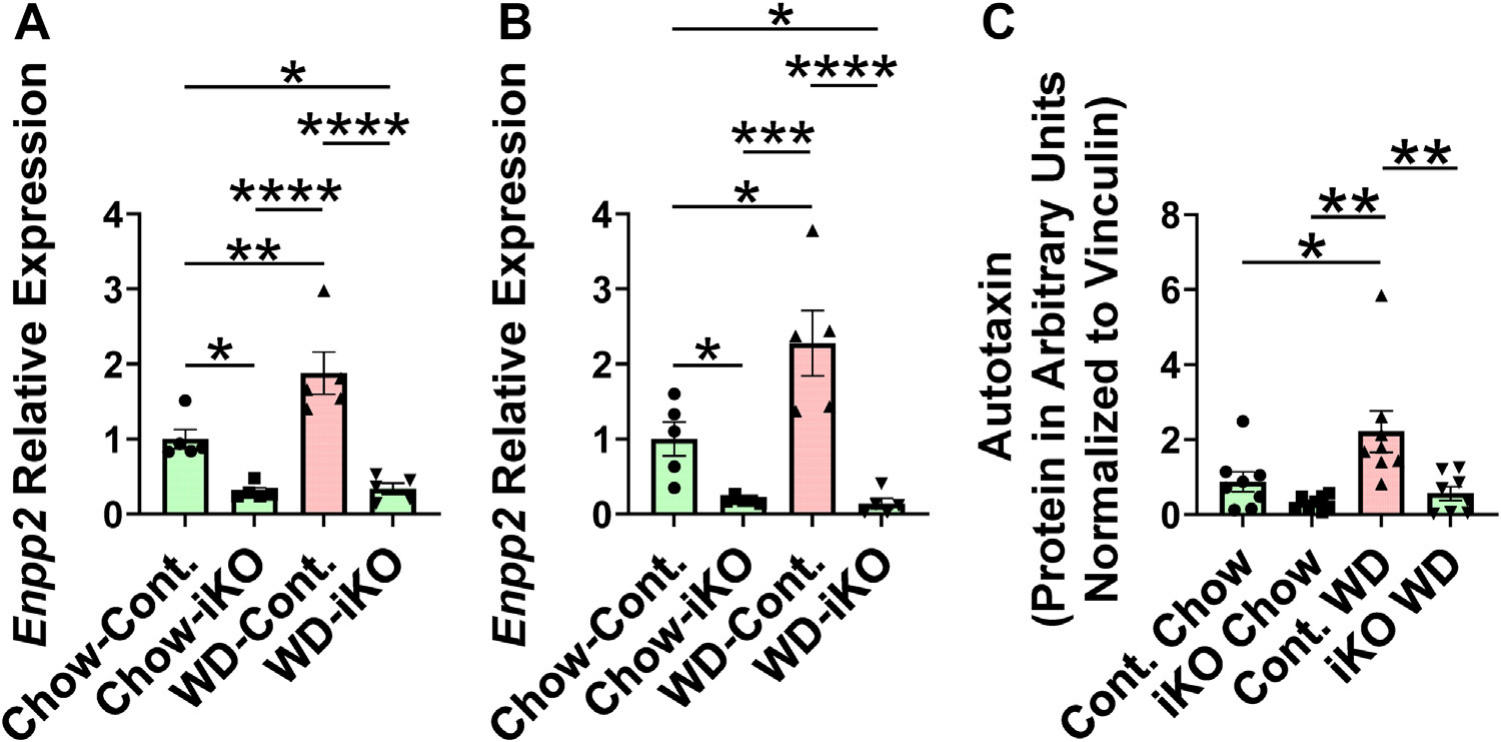
*Enpp2* gene expression and autotaxin protein levels in jejunum. A: *Enpp2* gene expression after feeding a chow diet (Chow) or WD for 2 weeks. Female *Enpp2*^*fl/fl*^*/Ldlr*^*−/−*^ (Cont.) mice 3.1 ± 0.4 months of age (n = 5 mice per group) and female *Enpp2*^*fl/fl*^*/Ldlr*^*−/−*^*/VilCre* (iKO) mice 3.9 ± 0.4 months of age (n = 5 mice per group) were fed either Chow or WD. After 2 weeks, enterocytes were isolated from the jejunum, and *Enpp2* gene expression was determined by RT-qPCR as described in the Materials and methods section. B: *Enpp2* gene expression after feeding Chow or WD for 5 months. Female Cont. mice, 2.6 ± 0.1 months of age (n = 5 mice per group) and female iKO mice 2.8 ± 0.1 months of age (n = 5 mice per group) were fed either Chow or WD. After 5 months, enterocytes were isolated from the jejunum, and *Enpp2* gene expression was determined by RT-qPCR as described in the Materials and methods section. C: Enterocyte autotaxin protein increased on WD in Cont. mice but not in iKO mice. Female Cont. mice or female iKO mice 4–5 months of age (n = 8 per group) were fed Chow or WD. After 2 weeks, enterocytes were prepared and highly purified by flow cytometry, the levels of autotaxin protein in the enterocytes were determined by Western blot analysis and normalized to vinculin as described in the Materials and methods section. ∗*P* < 0.05; ∗∗*P* < 0.01; ∗∗∗*P* < 0.001; ∗∗∗∗*P* < 0.0001.

The protein product of *Enpp2*, autotaxin, plays a major role in the generation of LPA (25). As shown in Fig. 1, gene expression for enterocyte *Enpp2* was markedly decreased in the iKO mice compared with the Cont. mice after 2 weeks on the diets (Fig. 1A) or after 5 months on the diets (Fig. 1B). At both time points in the Cont. mice, *Enpp2* expression increased on the WD compared with the chow diet (Fig. 1A, B). The residual *Enpp2* expression was likely because of nonenterocyte cells contaminating the preparations (26). Further purifying the enterocytes by flow cytometry followed by Western blotting demonstrated the increase in autotaxin protein on the WD compared with the chow diet in the Cont. mice (Fig. 1C). In contrast to jejunum enterocytes, hepatic expression of *Enpp2* was not altered in iKO mice confirming the specificity of the knockout (supplemental Fig. S3).

We recently reported that adding OxPLs ex vivo to jejunum from *Ldlr*^*−/−*^ mice fed a chow diet reproduced the changes in expression of genes that determine intestinal levels of antimicrobial peptides and proteins (22). In those studies, we demonstrated that adding the apoA-I mimetic peptide (6F) prevented these changes in gene expression (22). However, we did not study the expression of *Enpp2* in our previous studies (22). As shown in supplemental Fig. S4, adding OxPLs ex vivo to jejunum from *Ldlr*^*−/−*^ mice fed a chow diet increased gene expression for *Enpp2* similar to what was seen in vivo in Cont. mice fed the WD (Fig. 1). Moreover, adding the 6F peptide (but not a control peptide) prevented the increase in gene expression for *Enpp2* that was induced by the OxPLs (supplemental Fig. S4).

### LPA levels in jejunum enterocytes

**Fig. 2:**
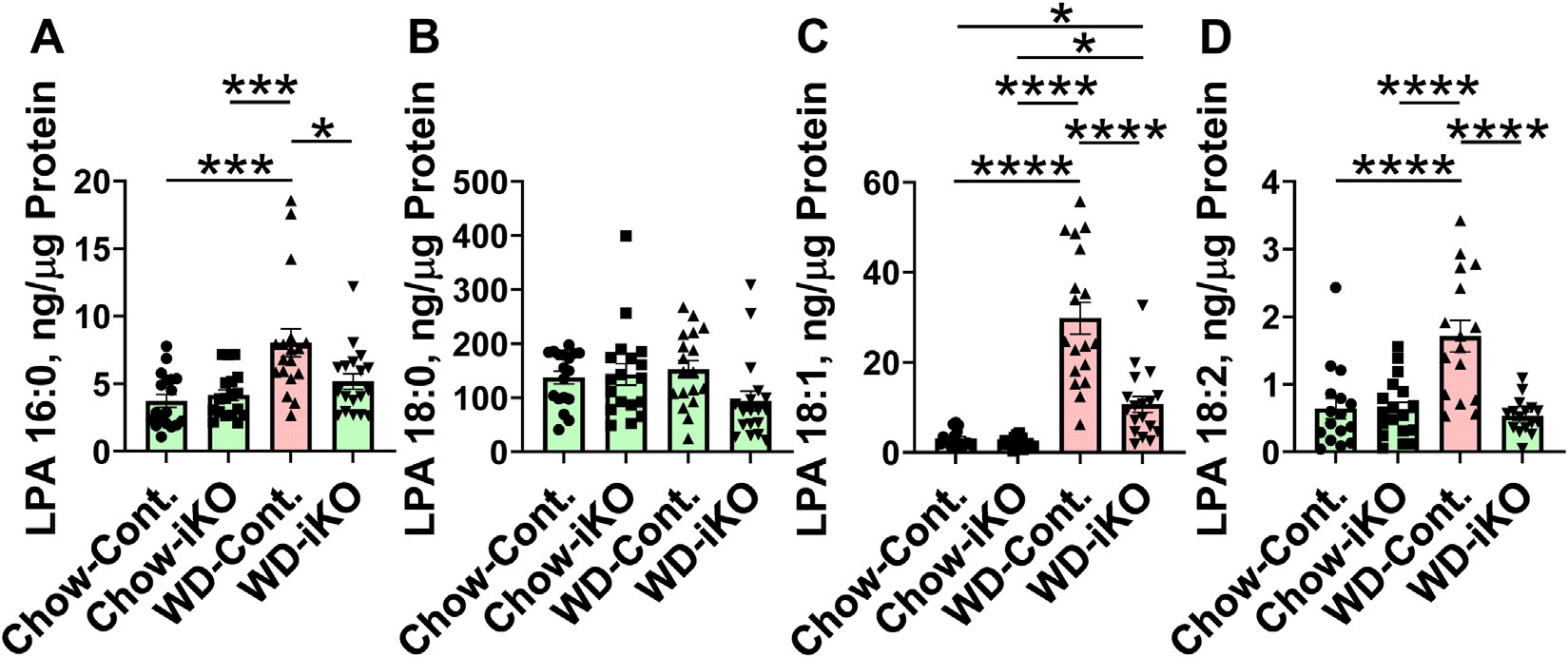
LPA levels in enterocytes. Male *Enpp2*^*fl/fl*^*/Ldlr*^*−/−*^ (Cont.) mice 6.1 ± 0.2 months of age (n = 18 mice per group) and male *Enpp2*^*fl/fl*^*/Ldlr*^*−/−*^*/VilCre* (iKO) mice 6.2 ± 0.1 months of age (n = 18 mice per group) were fed either the chow diet (Chow) or the WD. After 2 weeks, enterocytes were isolated from the jejunum, and LPA levels were determined as described in the Materials and methods section. A: LPA 16:0. B: LPA 18:0. C: LPA 18:1. D: LPA 18:2. ∗*P* < 0.05; ∗∗∗*P* < 0.001; ∗∗∗∗*P* < 0.0001.

On the chow diet, enterocyte levels for LPA 16:0 (Fig. 2A), LPA 18:0 (Fig. 2B), LPA 18:1(Fig. 2C), or LPA 18:2 (Fig. 2D) were not different in Cont. compared with iKO mice. On the WD in the Cont. mice, there was an increase in enterocyte levels for LPA 16:0, LPA 18:1, and LPA 18:2 compared with the chow diet; the levels for LPA 18:0 were not different by diet or genotype. In the iKO mice on the WD, the levels of LPA 16:0 and LPA 18:2 did not increase above those seen in mice on the chow diet. Enterocyte levels of LPA 18:1 increased on the WD in both Cont. and iKO mice, but the levels in the iKO mice were substantially less than in the Cont. mice on the WD.

### OxPL levels in jejunum mucus

**Fig. 3:**
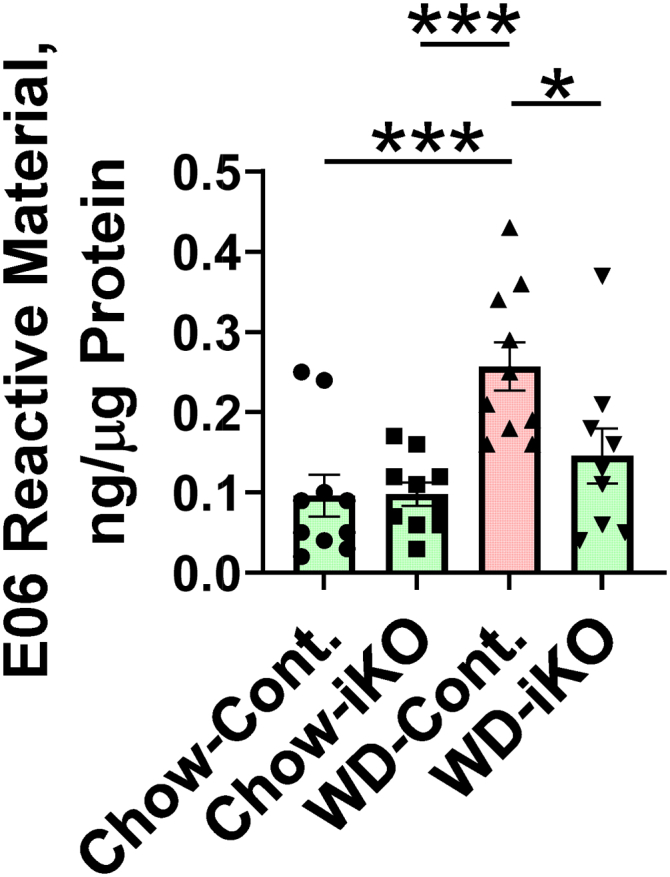
OxPLs in jejunum mucus. Female *Enpp2*^*fl/fl*^*/Ldlr*^*−/−*^ (Cont.) mice 3.5 ± 0.3 months of age (n = 10 mice per group) and female *Enpp2*^*fl/fl*^*/Ldlr*^*−/−*^*/VilCre* (iKO) mice 3.5 ± 0.3 months of age (n = 10 mice per group) were fed either the chow diet (Chow) or the WD. After 2 weeks, jejunum mucus was collected, and the levels of OxPLs were determined by E06 ELISA as described in the Materials and methods section. ∗*P* < 0.05; ∗∗∗*P* < 0.001.

The data from the Cont. mice shown in Fig. 3 confirm our previous report (22) regarding the increase in OxPLs in jejunum mucus from *Ldlr*^*−/−*^ mice fed a WD. Figure 3 also demonstrates that on the chow diet there was no difference in the level of OxPLs in jejunum mucus from Cont. or iKO mice. However, while the Cont. mice showed an increase in the levels of OxPLs in jejunum mucus on the WD, the levels of OxPLs in the mucus of the iKO mice on the WD were not significantly different from those seen in the chow-fed mice (Fig. 3). These data together with the data in Fig. 2, supplemental Fig. S2 and Fig. 1C are correlative suggesting that the increased levels of OxPLs in jejunum mucus in *Ldlr*^*−/−*^ mice fed a WD may be due, at least in part, to the local generation of LPA by enterocyte autotaxin.

### Gene expression in jejunum enterocytes for peptides and proteins that affect antimicrobial activity

**Fig. 4:**
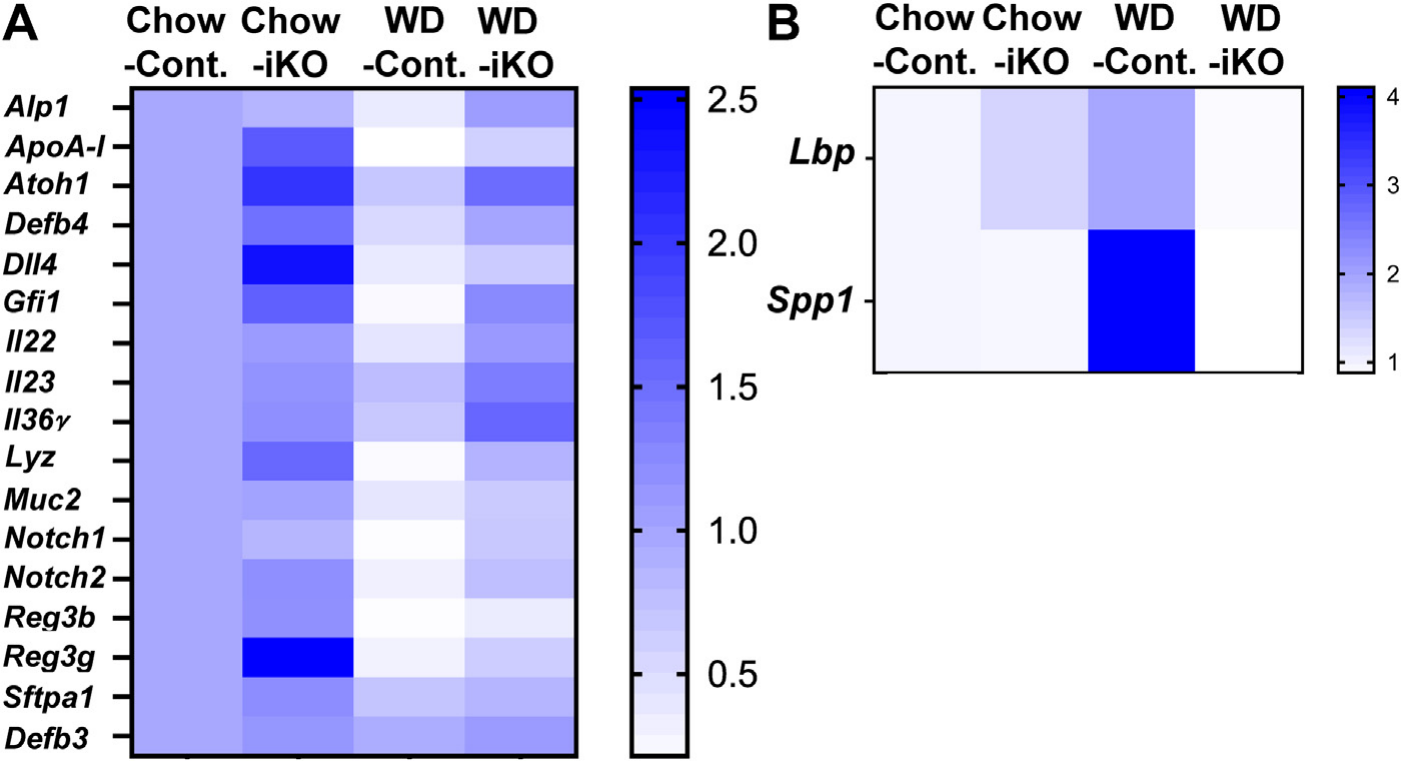
Nineteen genes that directly or indirectly regulate the levels of intestinal antimicrobial peptides and proteins were selected for study. Female *Enpp2*^*fl/fl*^*/Ldlr*^*−/−*^ (Cont.) mice 3.3 ± 0.3 months of age (n = 10 mice per group) and female *Enpp2*^*fl/fl*^*/Ldlr*^*−/−*^*/VilCre* (iKO) mice 3.4 ± 0.3 months of age (n = 10 mice per group) were fed either the chow diet (Chow) or the WD. After 2 weeks, enterocytes were isolated from the jejunum of the mice, and gene expression for these 19 genes was determined by RT-qPCR as described in the Materials and methods section. The data are presented as a heat map, which compares the expression of each gene to that in Cont. mice on a chow diet. A: The results are shown for 17 genes whose expression was previously reported to have decreased in the jejunum of *Ldlr*^*−/−*^ mice fed a WD (22): intestinal alkaline phosphatase (*Alp1*) (34, 35, 36, 37, 38, 39); apolipoprotein A-I (*ApoA*-I) (40, 41); atonal homolog 1 (*Atoh1*) (27, 28, 29); defensin 4 (*Defb4*) (42, 43); delta-like canonical notch ligand 4 (*Dll4*) (44); growth factor independent protein 1 (*Gfi1*) (30); interleukin 22 (*Il22*) (31, 45); interleukin 23 (*Il23*) (31, 46, 47); interleukin 36γ (*Il36γ*) (31); lysozyme (*Lyz*) (48); mucin 2 (*Muc2*) (49); notch receptor 1 (*Notch1*) (33); notch receptor 2 (*Notch2*) (31); regenerating islet-derived 3b (*Reg3b*) (50); regenerating islet-derived 3g (*Reg3g*) (51); surfactant A (*Sftpa1*) (52); and defensin 3 (*Defb3*) (53, 54). B: The results are shown for two genes whose expression was previously reported to have increased in the jejunum of *Ldlr*^*−/−*^ mice fed a WD (22): LPS-binding protein (*Lbp*) (55, 56); secreted phosphoprotein 1 (*Spp1*) (57, 58). Statistical analysis of the data in Fig. 4 is shown in supplemental Table S3.

We previously reported (22) that when *Ldlr*^*−/−*^ mice were fed a WD, the expression of 22 genes that directly or indirectly regulate the levels of intestinal antimicrobial peptides and proteins decreased, and the expression of two genes increased. Adding Tg6F to the WD reduced these changes (22). The protein products of many of these genes are produced in goblet and/or Paneth cells. Two important factors required for the formation of goblet and Paneth cells are the basic helix-loop-helix transcription factor atonal homolog 1 (*Atoh1*) that is also known as *Math1* (27, 28, 29), and growth factor independent protein 1 (*Gfi1*), a zinc-finger protein family member that is a direct target gene of *Atoh1*, and is required for the formation of Paneth cells (30). Notch pathway genes are also important for the production of intestinal antimicrobial peptides and proteins (31, 32, 33). Figure 4A and supplemental Table S3 present data for 17 of the 22 genes that previously showed decreased gene expression on the WD in *Ldlr*^*−/−*^ mice with wild-type *Enpp2* (22). Figure 4B and supplemental Table S3 present data for two genes that previously showed increased gene expression on the WD in *Ldlr*^*−/−*^ mice with wild-type *Enpp2* (22); LPS-binding protein (*Lbp*) and *Spp1* whose gene product is osteopontin. The data in Fig. 4A, B are presented as a heat map. Figure 4A confirms a decrease in gene expression in the jejunum of the Cont. mice fed a WD for 15 of the 17 genes that were previously seen to decrease on the WD (22). In the present studies, in contrast to the previous studies (22), gene expression for surfactant A (*Sftpa1*) and defensin 3 (*Defb3*) did not significantly change on the WD (Fig. 4A and supplemental Table S3). The data from the Cont. mice in Fig. 4B confirm our previous observation (22) that gene expression for *Lbp* and *Spp1* increased on the WD and demonstrate results similar to those reported (22) after adding Tg6F to the WD, that is, enterocyte-specific KO of *Ennp2* prevented the increased expression of these two genes on the WD. Seventeen of the 19 genes in Fig. 4A, B showed a significant difference between iKO mice that were fed the chow diet and Cont. mice that were fed the WD (supplemental Table S3). Ten of the 19 genes showed a significant difference between Cont. mice that were fed the WD and iKO mice that were fed the WD, and in one additional gene (*Defb4*), significance was almost achieved (*P* = 0.0560). In six genes in which the difference between Cont. mice that were fed the WD and iKO mice that were fed the WD did not reach statistical significance, gene expression in the Cont. mice fed the WD was significantly decreased compared with Cont. mice fed the chow diet (supplemental Table S3).

### Determination of peptide and protein levels that affect antimicrobial activity and quantification of the cells that produce these peptides and proteins

**Fig. 5:**
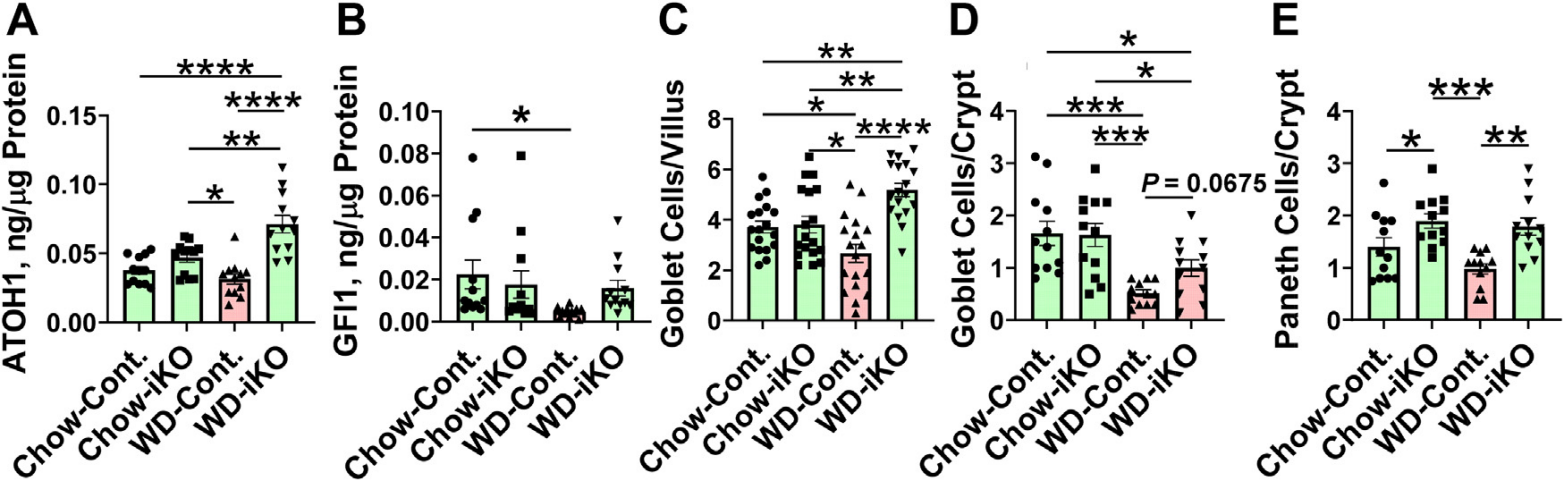
Quantification of atonal homolog 1 (ATOH1) and growth factor independent protein 1 (GFI1) by ELISA and quantification of goblet and Paneth cells in jejunum by immunohistochemistry. A: Female *Enpp2*^*fl/fl*^*/Ldlr*^*−/−*^ (Cont.) mice 3.3 ± 0.2 months of age (n = 12 mice per group) and female *Enpp2*^*fl/fl*^*/Ldlr*^*−/−*^*/VilCre* (iKO) mice 3.5 ± 0.2 months of age (n = 12 mice per group) were fed either the chow diet (Chow) or the WD. After 2 weeks, the levels of ATOH1 in jejunum were determined by ELISA as described in the Materials and methods section. B: Male Cont. mice 6.9 ± 0.5 months of age (n = 12 mice per group) and male iKO mice 7.0 ± 0.5 months of age (n = 12 mice per group) were fed either Chow or WD. After 2 weeks, the levels of GFI1 in jejunum were determined by ELISA as described in the Materials and methods section. C: Male Cont. mice 6 weeks of age (n = 3 mice per group) and male iKO mice 6 weeks of age (n = 3 mice per group) were fed either Chow or WD. After 2 weeks, the number of goblet cells/villus was determined as described in the Materials and methods section. D: The number of goblet cells/crypt was determined in the mice in (C) as described in the Materials and methods section. E: The number of Paneth cells/crypt was determined in the mice in (C) as described in *the*Materials and methods*se*ction. ∗*P* < 0.05; ∗∗*P* < 0.01; ∗∗∗*P* < 0.001; ∗∗∗∗*P* < 0.0001.

On the WD, protein levels for ATOH1 that is required for the formation of goblet and Paneth cells were significantly increased in the iKO mice (Fig. 5A). On the WD in Cont. mice, protein levels of GFI1, which is required for the formation of Paneth cells, were decreased, but there was no decrease in GFI1 in the iKO mice (Fig. 5B). By immunohistochemistry, the number of goblet cells/villus were increased in iKO mice on the WD compared with Cont. mice on the chow diet (Fig. 5C), and goblet cells/crypt showed a trend to less of a decrease in iKO mice on the WD compared with Cont. mice on the WD (Fig. 5D). Paneth cells in the iKO mice were increased compared with the Cont. mice on both the chow diet and the WD (Fig. 5E).

**Fig. 6:**
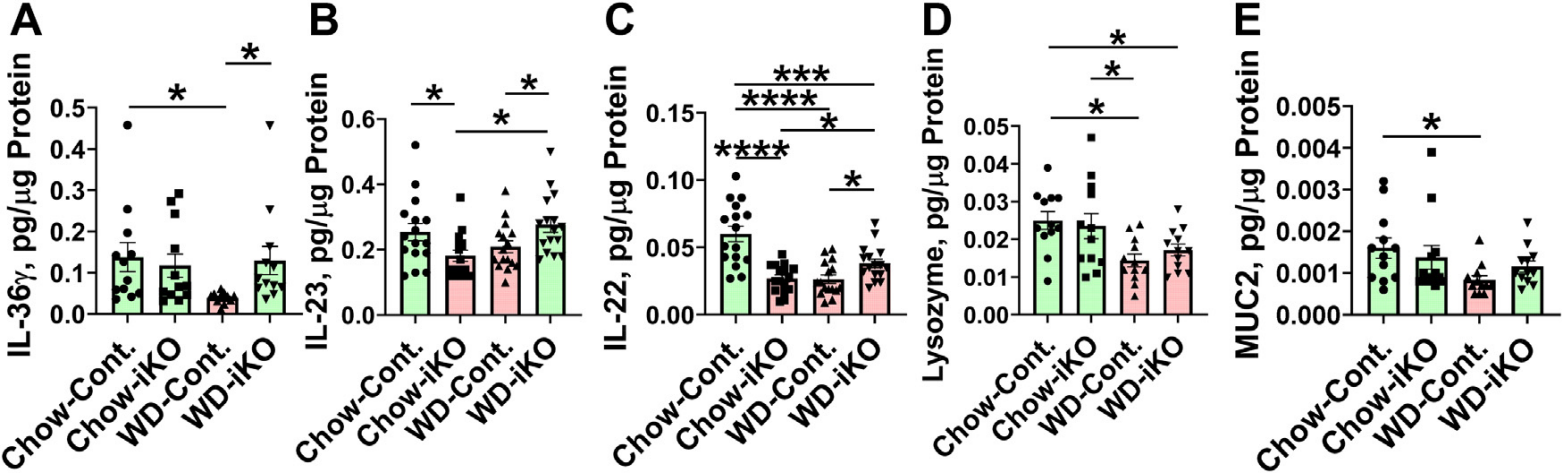
Quantification of interleukin 36γ (IL-36 γ), interleukin 23 (IL-23), interleukin 22 (IL-22), lysozyme and mucin2 (MUC2) by ELISA. A: IL-36 γ and B: IL-23 levels in the jejunums of the mice described in Fig. 5B (male *Enpp2*^*fl/fl*^*/Ldlr*^*−/−*^ [Cont.] mice 6.9 ± 0.5 months of age [n = 12 mice per group] and male *Enpp2*^*fl/fl*^*/Ldlr*^*−/−*^*/VilCre* [iKO] mice 7.0 ± 0.5 months of age [n = 12 mice per group]) were fed either the chow diet (Chow) or the WD. After 2 weeks, the levels of IL-36 γ and IL-23 were determined by ELISA as described in the Materials and methods section. C: Male Cont. mice 6.5 ± 0.4 months of age (n = 16 mice per group) and male iKO mice 6.9 ± 0.5 months of age (n = 16 mice per group) were fed either Chow or WD. After 2 weeks, the levels of IL-22 in jejunum were determined by ELISA as described in the Materials and methods section. D: Lysozyme and (E) MUC2 levels in jejunum mucus from the mice in (A and B) were determined by ELISA as described in th*e*Materials and methods*s*ection. ∗*P* < 0.05; ∗∗∗*P* < 0.001; ∗∗∗∗*P* < 0.0001.

Figure 6 shows that on the WD in the Cont. mice compared with iKO mice, protein levels in the jejunum were decreased for IL-36γ (Fig. 6A), IL-23 (Fig. 6B), and IL-22 (Fig. 6C), which are critical for antimicrobial activity in the intestine (22). In each instance, these changes were prevented or the decrease was less in the iKO mice on the WD. On the chow diet, both IL-23 and IL-22 were decreased in the iKO mice (Fig. 6B, C), which suggests that the control of IL-23 and IL-22 levels may be more complex than that for IL-36γ. On the WD, lysozyme protein levels in jejunum mucus were decreased in both Cont. and iKO mice (Fig. 6D) indicating that *Enpp2* knockout in enterocytes did not improve the WD-mediated decrease in lysozyme protein levels. On the WD in the Cont. mice (but not in the iKO mice), protein levels in jejunum mucus were decreased for the major intestinal mucus protein, MUC2 (Fig. 6E).

**Fig. 7:**
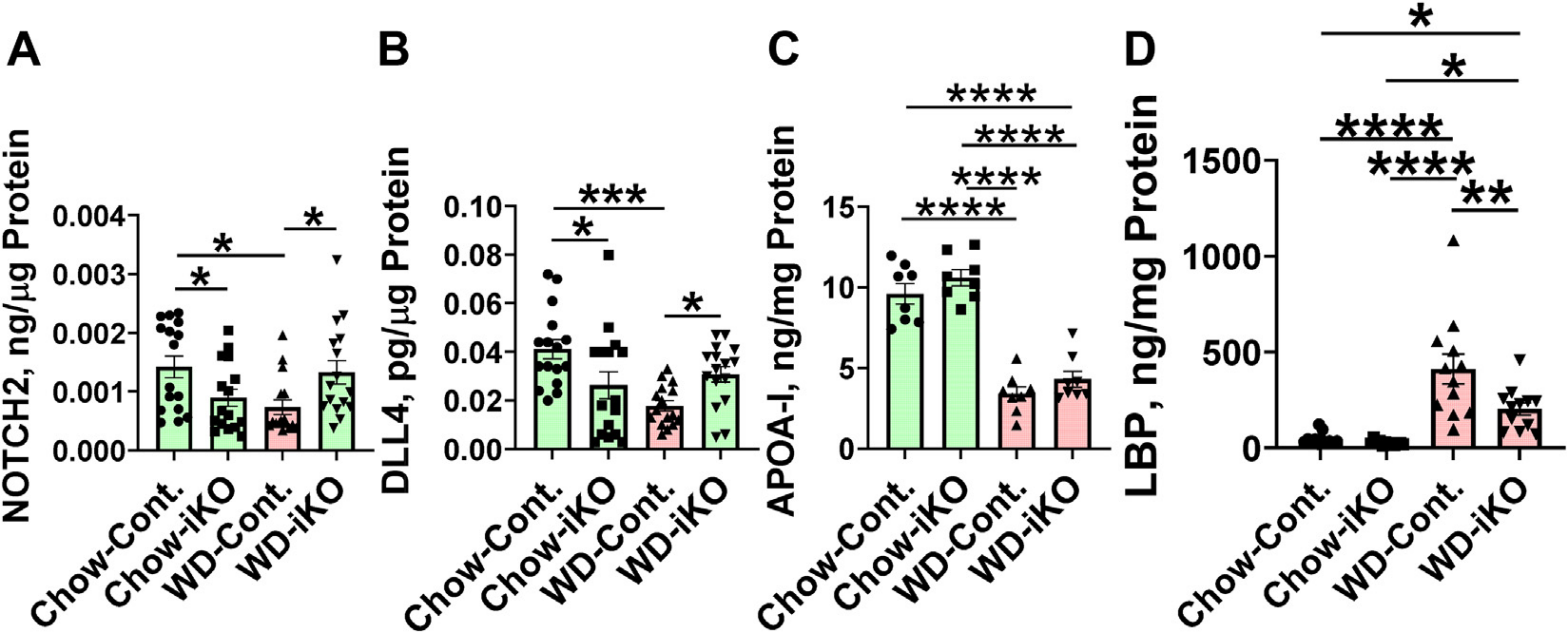
Quantification of NOTCH2, DLL4, APOA-I, and LBP by ELISA. Male *Enpp2*^*fl/fl*^*/Ldlr*^*−/−*^ (Cont.) mice 6.5 ± 0.4 months of age (n = 16 mice per group) and male *Enpp2*^*fl/fl*^*/Ldlr*^*−/−*^*/VilCre* (iKO) mice 6.9 ± 0.5 months of age (n = 16 mice per group) were fed either the chow diet (Chow) or the WD. After 2 weeks, jejunum levels of NOTCH2 (A) or DLL4 (B) were determined by ELISA as described in the Materials and methods section. C: Male Cont. mice 7.4 ± 0.3 months of age (n = 8 mice per group) and male iKO mice 7.4 ± 0.3 months of age (n = 8 mice per group) were fed either Chow or WD. After 2 weeks, the levels of APOA-I in jejunum mucus were determined by ELISA as described in the Materials and methods section. D: Female Cont. mice 3.4 ± 0.3 months of age (n = 12 mice per group) and female iKO mice 3.3 ± 0.3 months of age (n = 12 mice per group) were fed either Chow or WD. After 2 weeks, the levels of LBP in jejunum mucus were determined by ELISA as described in the Materials and methods section. ∗*P* < 0.05; ∗∗*P* < 0.01; ∗∗∗*P* < 0.001; ∗∗∗∗*P* < 0.0001.

Figure 7 demonstrates that on the WD in the Cont. mice, protein levels in the jejunum were decreased for NOTCH2 (Fig. 7A) and the Notch ligand DLL4 (Fig. 7B); these WD-mediated changes were prevented in the iKO mice. On the WD in both the Cont. and iKO mice, APOA-I protein levels were decreased in jejunum mucus (Fig. 7C). On the WD in the Cont. mice, jejunum mucus levels for LBP increased, but the increase was much less in the iKO mice (Fig. 7D).

### LPS levels increased in jejunum mucus on the WD but increased much less in iKO mice

**Fig. 8:**
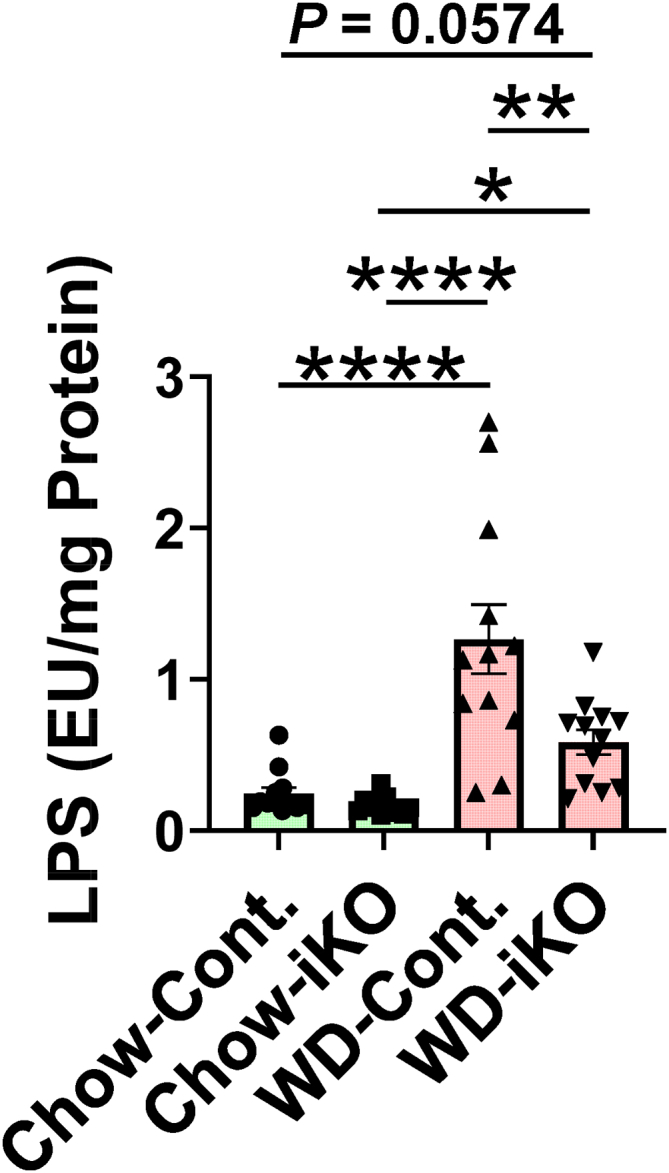
LPS levels increased in jejunum mucus on the WD but increased much less in the *Enpp2*^*fl/fl*^*/Ldlr*^*−/−*^*/VilCre* (iKO) mice. Female *Enpp2*^*fl/fl*^*/Ldlr*^*−/−*^ (Cont.) mice 3.3 ± 0.3 months of age (n = 12 mice per group) and female iKO mice 3.4 ± 0.3 months of age (n = 12 mice per group) were fed either the chow diet (Chow) or WD. After 2 weeks, LPS levels were determined in jejunum mucus as described in the Materials and methods section. ∗*P* < 0.05; ∗∗*P* < 0.01; ∗∗∗∗*P* < 0.0001.

LPS levels increased in jejunum mucus on the WD compared with the chow diet. On the WD, LPS levels in iKO mucus increased much less than in Cont. mucus (Fig. 8). Supplemental Fig. S5 demonstrates that the LPS content of the chow diet was substantially higher than that of the WD indicating that the increased levels of LPS in jejunum mucus on the WD were not because of LPS in the diet.

### Plasma total cholesterol, triglycerides, and apoA-I levels after 2 weeks on chow or WD

**Fig. 9:**
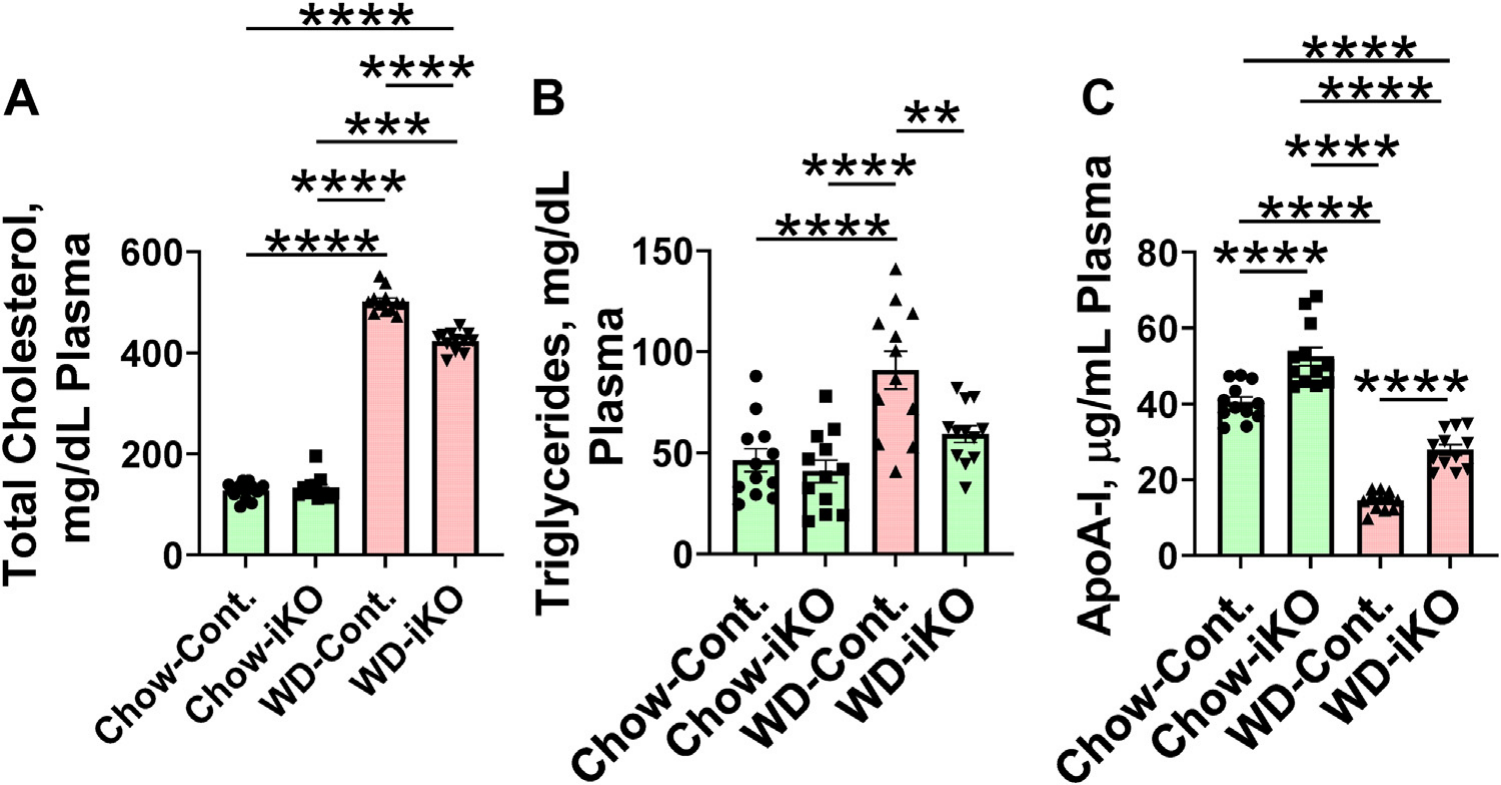
Plasma total cholesterol, triglycerides, and apoA-I after 2 weeks on the chow diet (Chow) or the WD. The mice described in Fig. 5A (female *Enpp2*^*fl/fl*^*/Ldlr*^*−/−*^ (Cont.) mice 3.3 ± 0.2 months of age [n = 12 mice per group] and female *Enpp2*^*fl/fl*^*/Ldlr*^*−/−*^*/VilCre* [iKO] mice 3.5 ± 0.2 months of age [n = 12 mice per group]) were fed either Chow or WD. After 2 weeks, plasma levels of total cholesterol (A), triglycerides (B), and apoA-I (C) were determined as described in the Materials and methods section. ∗∗*P* < 0.01; ∗∗∗*P* < 0.001; ∗∗∗∗*P* < 0.0001.

As shown in Fig. 9 after 2 weeks on the chow diet, there was no difference between the levels of plasma total cholesterol (Fig. 9A) or plasma triglycerides (Fig. 9B) in Cont. compared with iKO mice. However, plasma apoA-I levels were higher in iKO mice compared with Cont. mice on the chow diet (Fig. 9C). Plasma total cholesterol levels increased in both Cont. and iKO mice on the WD but increased less in the iKO mice (Fig. 9A). Plasma triglyceride levels increased in the Cont. mice on the WD but did not increase in iKO mice (Fig. 9B). Plasma apoA-I levels decreased on the WD in both Cont. and iKO mice but decreased less in the iKO mice compared with the Cont. mice (Fig. 9C). On the chow diet, HDL-cholesterol levels were higher in iKO mice. On the WD, HDL-cholesterol levels were decreased in both Cont. and iKO mice but decreased less in the iKO mice (supplemental Fig. S6).

### Plasma LPA levels after 2 weeks on chow diet or WD

**Fig. 10:**
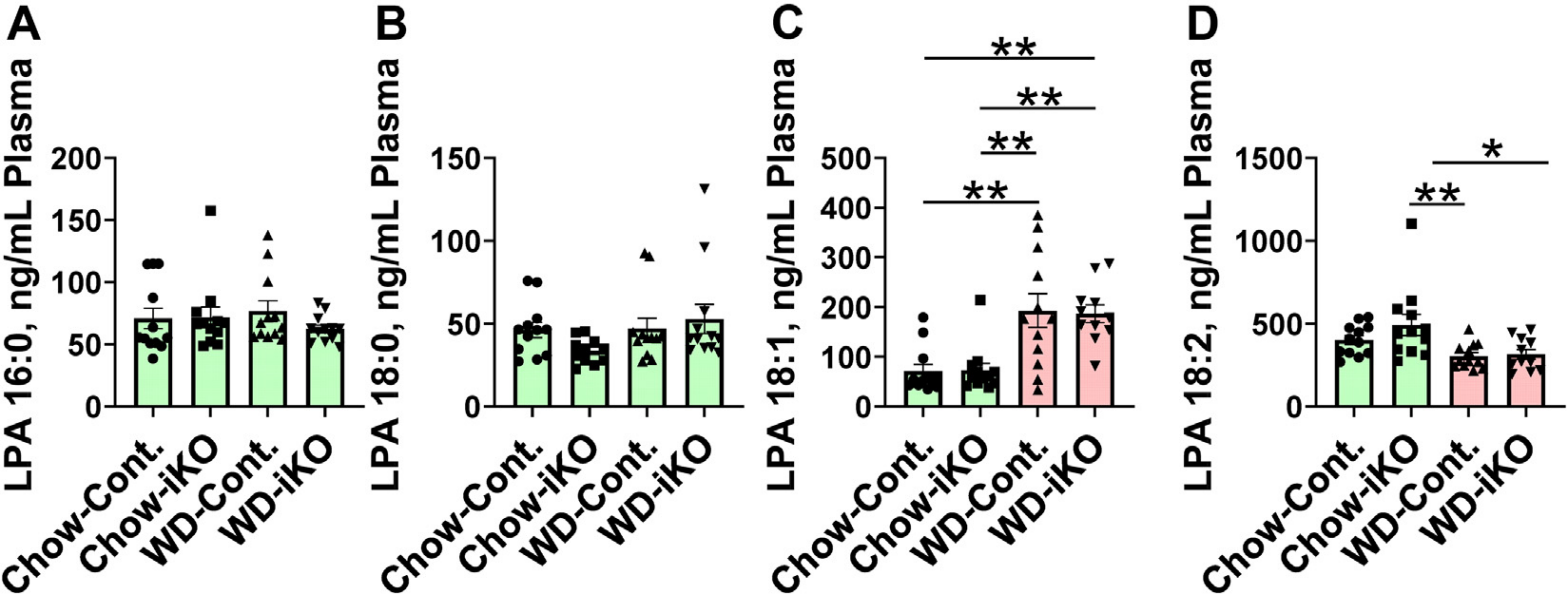
Plasma LPA levels after 2 weeks on the chow diet (Chow) or the WD. The mice described in Fig. 5A (female *Enpp2*^*fl/fl*^*/Ldlr*^*−/−*^ [Cont.] mice 3.3 ± 0.2 months of age [n = 12 mice per group] and female *Enpp2*^*fl/fl*^*/Ldlr*^*−/−*^*/VilCre* [iKO] mice 3.5 ± 0.2 months of age [n = 12 mice per group]) were fed either Chow or WD. After 2 weeks, plasma levels of LPA 16:0 (A), LPA 18:0 (B), LPA 18:1 (C), and LPA 18:2 (D) were determined as described in the Materials and methods section. ∗*P* < 0.05; ∗∗*P* < 0.01.

Figure 10 demonstrates that in contrast to jejunum LPA levels, there was no genotype effect in plasma after 2 weeks on the chow diet or WD. Moreover, after 2 weeks on the diets, there was no change in the levels of plasma LPA 16:0 (Fig. 10A) or LPA 18:0 (Fig. 10B) on the WD compared with the chow diet. Both Cont. and iKO mice had increased levels of LPA 18:1 in plasma on the WD compared with the chow diet, but in contrast to the case in jejunum where the iKO mice had less of an increase on the WD (Fig. 2C), there was no difference in plasma LPA 18:1 levels (Fig. 10C). Plasma levels of LPA 18:2 on the WD decreased compared with chow, and there was no difference between Cont. and iKO mice (Fig. 10D), which was distinctly different from the case in the jejunum where LPA 18:2 increased on the WD in Cont. mice compared with the chow diet but remained the same as in chow-fed mice in the iKO mice on the WD (Fig. 2D).

### Plasma LBP, LPS, IL-6, and SAA after 2 weeks on chow diet or WD

**Fig. 11:**
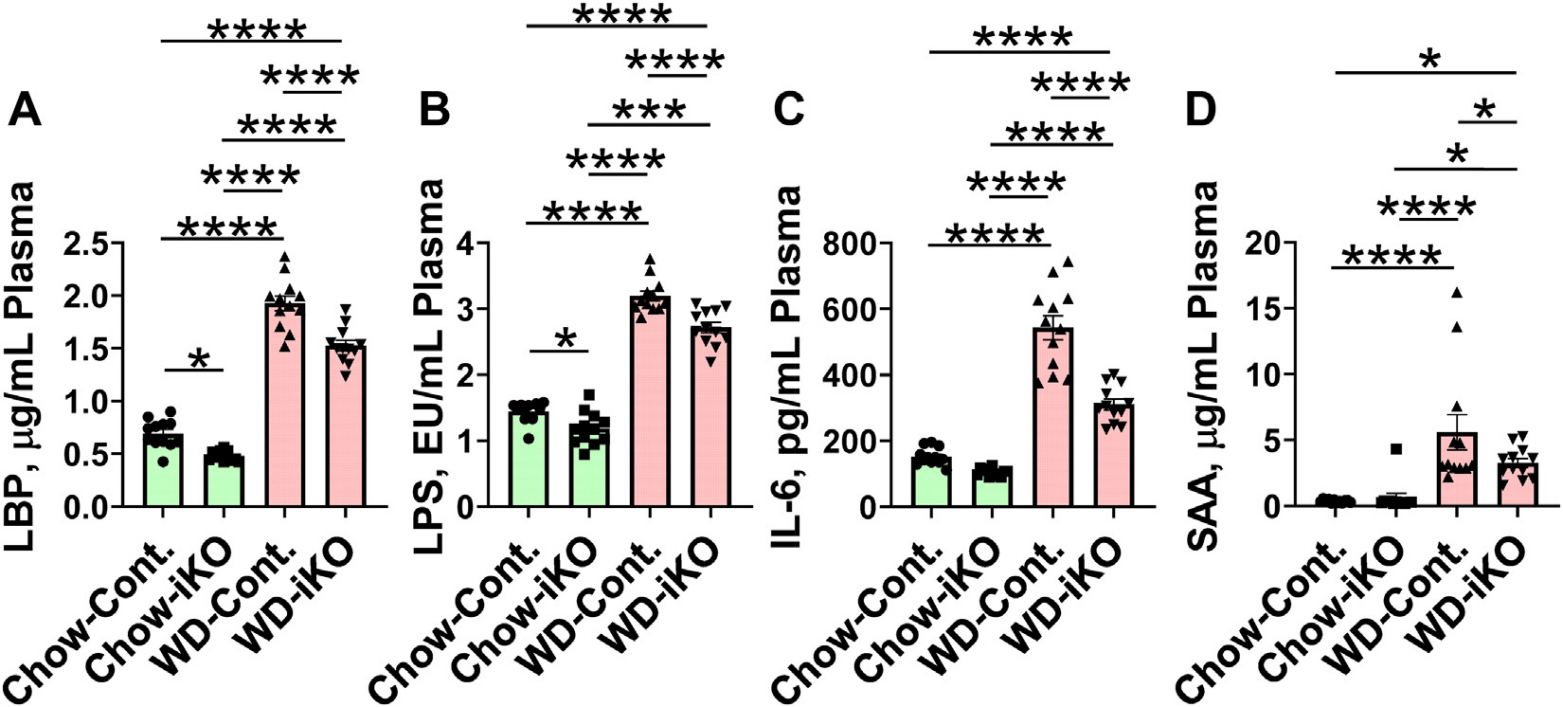
Plasma levels of LBP, LPS, IL-6, and SAA after 2 weeks on the chow diet (Chow) or the WD. The mice described in Fig. 5A (female *Enpp2*^*fl/fl*^*/Ldlr*^*−/−*^ [Cont.] mice 3.3 ± 0.2 months of age [n = 12 mice per group] and female *Enpp2*^*fl/fl*^*/Ldlr*^*−/−*^*/VilCre* [iKO] mice 3.5 ± 0.2 months of age [n = 12 mice per group]) were fed either Chow or WD. After 2 weeks, plasma levels of LBP (A), LPS (B), IL-6 (C), and SAA (D) were determined as described in the Materials and methods section. ∗*P* < 0.05; ∗∗∗*P* < 0.001; ∗∗∗∗*P* < 0.0001.

As shown in Fig. 11, plasma levels of LBP and LPS increased on the WD compared with the chow diet, and on both chow diet and the WD, there were lower plasma levels of LBP and LPS in iKO mice compared with Cont. mice (Fig. 11A, B, respectively). Paralleling these changes, plasma levels of IL-6 and SAA were higher in mice on the WD compared with the chow diet, and the levels were lower in iKO mice on the WD compared with Cont. mice (Fig. 11C, D, respectively).

### Changes after 5 months on chow diet or WD

The levels of plasma total cholesterol, triglycerides, and apoA-I after feeding the diets for 5 months are shown in supplemental Fig. S7. The changes in plasma cholesterol, triglycerides, and apoA-I after 5 months on the diets were similar to the changes after 2 weeks, except that the plasma cholesterol levels in the iKO mice on the WD (345 ± 24 mg/dl) compared with Cont. mice on chow (267 ± 14) did not quite reach statistical significance (*P* = 0.0712).

Plasma LPA levels are shown in supplemental Fig. S8. Plasma LPA 16:0 and LPA 18:0 levels after feeding the diets for 5 months were lower in the mice receiving WD compared with mice receiving chow but were not different between Cont. and iKO mice. After 5 months, plasma levels of LPA 18:1 remained higher on WD compared with chow, but in contrast to the case at 2 weeks, they were lower in the iKO mice on WD compared with Cont. mice on WD. After 5 months, LPA 18:2 levels remained lower on the WD compared with chow, and they were significantly lower in the iKO mice on WD compared with Cont. mice on WD.

**Fig. 12:**
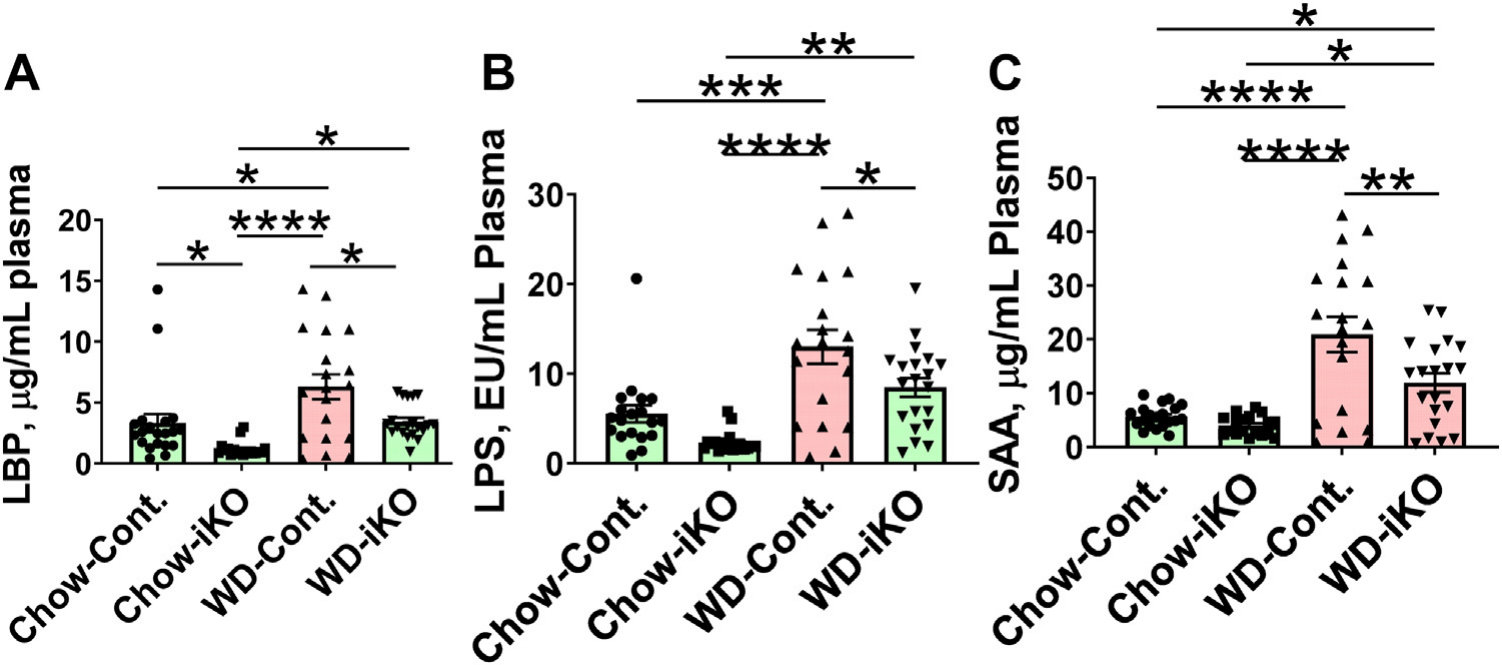
Plasma LBP, LPS, and SAA after 5 months on the chow diet (Chow) or the WD. The mice described in supplemental Fig. S7 (female *Enpp2*^*fl/fl*^*/Ldlr*^*−/−*^ [Cont.] mice 2.6 ± 0.1 months of age [n = 19–20 mice per group] and female *Enpp2*^*fl/fl*^*/Ldlr*^*−/−*^*/VilCre* [iKO] mice 2.8 ± 0.1 months of age [n = 18–20 mice per group]) were fed either Chow or WD. After 5 months, plasma levels of LBP (A), LPS (B), and SAA (C) were determined as described in the Materials and methods section. ∗*P* < 0.05; ∗∗*P* < 0.01; ∗∗∗*P* < 0.001; ∗∗∗∗*P* < 0.0001.

Plasma levels of LBP, LPS, and SAA are shown in Fig. 12. On the chow diet, plasma levels of LBP were lower in the iKO mice compared with the Cont. mice (Fig. 12A). Plasma levels of LBP increased after 5 months on the WD compared with the chow diet in Cont. mice but were not different from chow in iKO mice on the WD (Fig. 12A). Plasma LPS levels increased after 5 months on the WD in Cont. mice, but in iKO mice on the WD, plasma LPS levels were not different from Cont. mice on the chow diet (Fig. 12B). SAA levels increased after 5 months on the WD and increased less in the iKO mice (Fig. 12C).

**Fig. 13:**
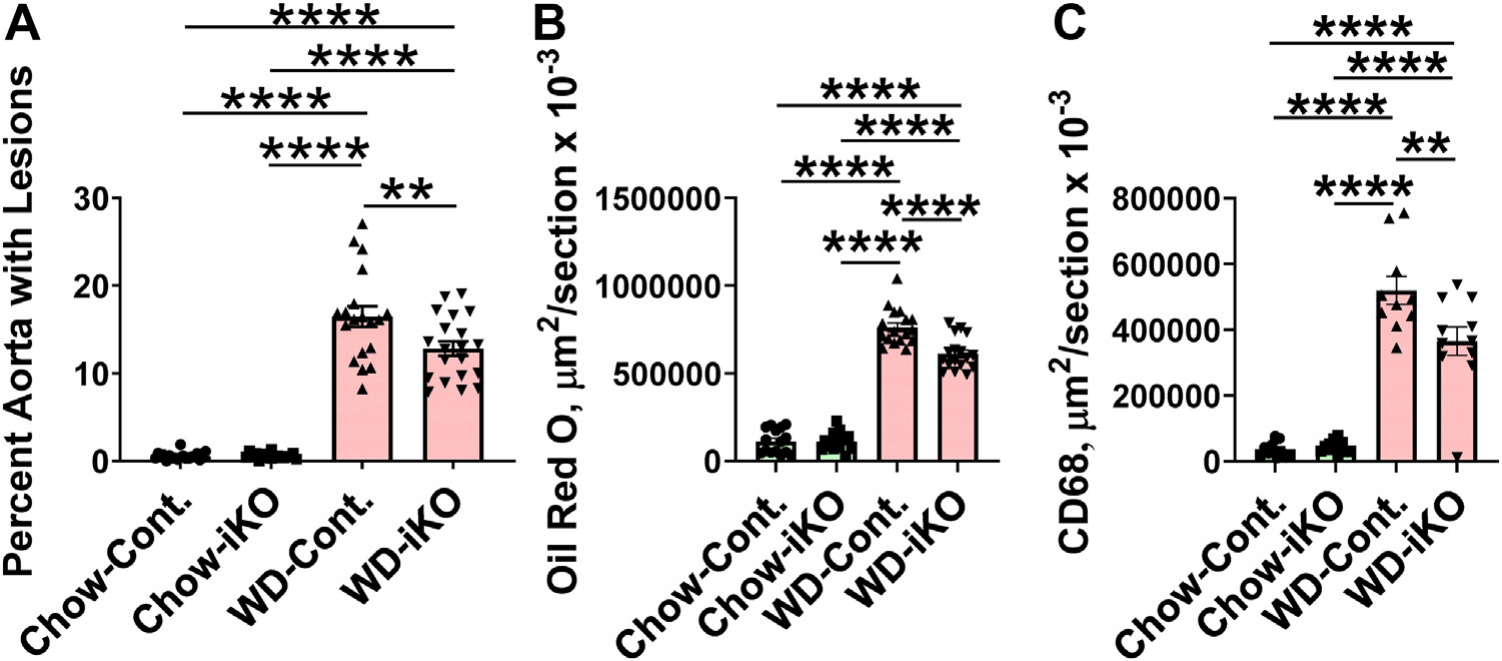
Aortic lesions after 5 months on the chow diet (Chow) or the WD. The mice described in supplemental Fig. S7 (female *Enpp2*^*fl/fl*^*/Ldlr*^*−/−*^ [Cont.] mice 2.6 ± 0.1 months of age [n = 19–20 mice per group] and female *Enpp2*^*fl/fl*^*/Ldlr*^*−/−*^*/VilCre* [iKO] mice 2.8 ± 0.1 months of age [n = 18–20 mice per group]) were fed either Chow or WD. After 5 months, the extent of aortic atherosclerosis was determined as the percent of aorta with lesions (A), or the lesion area in the aortic root was determined by Oil Red O staining (B), or the area staining for CD68 was determined (C) as described in the Materials and methods section. ∗∗*P* < 0.01; ∗∗∗∗*P* < 0.0001.

After 5 months on the WD, there was less of an increase in the iKO mice for the following parameters: percent of whole aorta with atherosclerotic lesions (Fig. 13A); aortic root Oil Red O lesion area (Fig. 13B); and area positive for macrophages (CD68) (Fig. 13C). As shown in supplemental Fig. S9, plasma levels of LPS significantly correlated with the data in Fig. 13.

## Discussion

The epithelium of the intestine turns over every ∼3–5 days. Thus, the proliferation and differentiation of intestinal epithelial cells is critical for maintenance of the epithelial barrier in the intestine. Konno *et al.* (9) demonstrated that LPA 18:1 plays an important role in the proliferation and differentiation of intestinal epithelial cells. Unsaturated LPA species including LPA 18:1 are the preferred ligands for LPA receptors 1, 2, 3, 4, 5, GPR87, P2Y5, P2Y10, and GPR35 (59). Lin *et al.* (10, 11) demonstrated that LPA receptor 1 is important for intestinal epithelial homeostasis, wound closure, intestinal epithelial barrier function including preventing the entry of gut bacteria, and susceptibility to colitis. The LPA species that Yun *et al.* (11) used in their experiments was also LPA 18:1. Orally administering LPA 18:1 as in the current study or LPA 18:2 (13, 14) at a dose of 1 μg per mg chow resulted in LPA levels in the small intestine of mice that were similar to those achieved by feeding a WD and mimicked many of the changes induced by the WD including causing aortic atherosclerosis that was qualitatively the same as that seen on the WD (14). The levels of unsaturated phospholipids in enterocytes on the WD are largely not derived from unsaturated phospholipids in the WD. Indeed, we found that the chow diet contained dramatically more unsaturated phospholipids compared with the WD, especially 18:1 containing phospholipids (16). The WD mainly contains saturated fatty acids; it induces the expression of *Scd1* (18) and *Lpcat3* in enterocytes (14), which provide the unsaturated phospholipid substrates that are acted upon by enterocyte autotaxin, and likely accounts for most of the increased unsaturated LPA in enterocytes from mice on the WD.

Similar to our reports, Zhou *et al.* (4) reported that unsaturated LPA but not saturated LPA (18:0) accelerated the progression of atherosclerosis in mice. In our previous studies adding Tg6F to the WD reduced the levels of these LPA species in the jejunum and reduced the WD-induced changes (13, 14). In the current studies, the response to the WD by the iKO mice was remarkably similar to that previously reported in *Ldlr*^*−/−*^ mice that were wild type for *Enpp2* that were fed a WD that was supplemented with Tg6F (22). When administered orally, the 6F peptide is not absorbed intact but is found intact in the lumen of the small intestine (12). Therefore, after oral administration, it must act in the vicinity of the enterocytes. The apoA-I mimetic peptide 6F is known to bind OxPLs such that they can no longer interact with cells (23). Supplemental Fig. S4 shows that adding OxPLs to jejunum ex vivo induced *Enpp2* expression, which was prevented by adding the 6F peptide. The 6F peptide is one of three class A 18-amino acid residue apoA-I mimetic peptides (4F, 5F, and 6F) that bind a number of lipids with much higher affinity than native apoA-I (23, 24). Unsaturated LPA is among the lipids bound by this class of apoA-I mimetic peptides with extraordinarily high affinity (60). Therefore, it is possible that oral 6F acts in part by binding and reducing the levels of both LPA and OxPLs in enterocytes.

The results in Fig. 1 demonstrating that the WD induced the expression of *Enpp2* in enterocytes in Cont. mice are similar to the findings of Dusaulcy *et al.* (61) showing that expression of *Enpp2* in subcutaneous, perigonadal, and perirenal adipose tissue was significantly increased in *Enpp2* wild-type mice fed a high-fat diet compared with when these mice were fed standard mouse chow. However, Dusaulcy *et al.* (61) did not see an increase in expression of *Enpp2* in brown adipose tissue or brain or kidney on the high-fat diet, demonstrating that local factors and specific tissue characteristics determine *Enpp2* expression. Dusaulcy *et al.* (61) did not measure the levels of OxPLs in their studies. They did see a 38% decrease in total plasma LPA levels on knockout of adipose tissue-specific *Enpp2* in chow-fed control mice, and there was a 62% increase in total plasma LPA levels on the high-fat diet compared with the chow diet in control mice but not in the adipose-specific knockout mice. They did not measure individual LPA species. In our studies, after 2 weeks, only LPA 18:1 levels increased in plasma on the WD compared with the chow diet, and there was no difference between the response of Cont. and iKO mice. However, after 2 weeks of feeding the diets, the plasma data were quite different from the enterocyte data. After 2 weeks, Cont. enterocyte levels of LPA 16:0, LPA 18:1, and LPA 18:2 were increased on the WD and were significantly less in the iKO enterocytes indicating that the changes in the intestine at this time point were not reflected in the plasma. In contrast to the results after 2 weeks of feeding the diets, after feeding the diets for 5 months, the iKO mice on the WD had significantly lower levels of plasma LPA 18:1 and LPA 18:2 compared with Cont. mice on WD. Dusaulcy *et al.* (61) measured plasma LPA levels after 13 weeks on the diets. Thus, periods longer than 2 weeks may be required for tissue-specific knockouts of autotaxin to be reflected in plasma LPA levels. Future studies measuring plasma autotaxin levels together with plasma LPA levels as a function of the time that the diets are fed will be required to determine if this is the case.

Brandon *et al.* (62) generated a different adipose-specific knockout of *Enpp2* than the one used by Dusaulcy *et al.* (61). Kraemer *et al.* (63) used the adipose-specific knockout of *Enpp2* that was generated by Brandon *et al.* (62) and found that it prevented the increase in LPA associated with LDL in genetically hyperlipidemic mouse models. We did not measure LPA levels in LDL.

We previously reported that adding unsaturated LPC to standard mouse chow qualitatively mimicked many of the features of feeding a WD to *Ldlr*^*−/−*^ mice (14) and was similar to adding unsaturated LPA to standard mouse chow (13). In those studies (14), adding an inhibitor of autotaxin (PF8380) partially prevented the ability of unsaturated LPC added to mouse chow to mimic the WD. Since PF8380 is absorbed and acts in many tissues, the role of the intestine could not be determined (14). The current studies unequivocally demonstrate that LPA generated locally in the intestine plays an important role in the WD-mediated uptake of gut-derived LPS, systemic inflammation, and enhanced atherosclerosis in *Ldlr*^*−/−*^ mice.

Lin *et al.* (64) used inducible whole-body deletion of *Enpp2* in adult mice to study colitis. They reported that whole-body deletion of *Enpp2* suppressed experimental colitis, and that B cells were a major source of autotaxin in the colon. Kim *et al.* (65) reported that macrophages from myeloid cell lineage-restricted *Enpp2* knockout mice had reduced Toll-like receptor 4 complex formation that was associated with attenuation of phagocytosis and inducible nitric oxide synthase expression. These mice exhibited increased bacterial content in intestinal mucosa, and when bred with global *Il10*^*−/−*^ mice on a C57BL/6 background for at least eight generations, they exhibited accelerated colitis. The authors concluded that myeloid cell lineage autotaxin deficiency compromises innate immune responses, thereby promoting microbe-associated gut inflammation (65). These authors did not use this model to study atherosclerosis.

Karshovska *et al.* (66) reported that a tamoxifen-induced endothelial cell-specific *Enpp2* knockout decreased the following: atherosclerosis plaque area, macrophages in lesions, monocyte adhesion, and endothelial expression of CXCL1 in male and female *Apoe*^*−/−*^ mice. In vitro, they found that *Enpp2* mediated mildly oxidized LDL-induced expression of CXCL1 in aortic endothelial cells by generating LPA 20:4, LPA 16:0, and LPA 18:1. Autotaxin was detected on the endothelial surface by confocal imaging. The expression of endothelial *Enpp2* was strongly correlated with plasma levels of LPA 16:0, LPA 18:0, and LPA 18:1 and aortic plaque size. These authors concluded that endothelial autotaxin promotes atherosclerosis and endothelial inflammation in a sex-independent manner that might be due to the generation of LPA 20:4, LPA 16:0, and LPA 18:1 from mildly oxidized lipoproteins located on the endothelial surface (66). We previously demonstrated that the biologic activity of mildly oxidized LDL was largely because of its content of OxPLs (67, 68, 69, 70). Karshovska *et al.* (66) did not report on OxPLs in their study.

The limitations of the data reported here included the following. In every experiment, mice of the same gender were compared (i.e., males and females were not mixed in any experiment), and each group being compared was closely matched for age. The data from these studies demonstrate that both male and female mice showed changes consistent with a role of enterocyte *Enpp2* in the parameters measured. However, because we used either male mice or female mice in each experiment (but not both in the same experiment), our data do not provide a quantitative comparison of male and female mice for all the parameters measured, and therefore, we cannot exclude that there may be quantitative gender-based differences for some of the parameters measured. Similarly, although the ages of the mice used in these studies were carefully matched in every experiment, we did not measure every parameter by age in both males and females, and therefore, we can neither exclude the possibility that there may be age-based differences for some of the parameters nor can we provide a definitive picture of how each parameter changes with age.

**Fig. 14:**
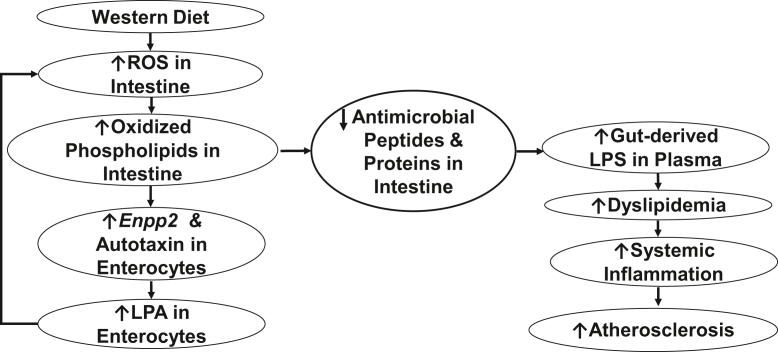
A schematic diagram showing that *i*) feeding a WD to *Ldlr*^*−/−*^ mice increases reactive oxygen species (ROS) in intestine that *ii*) promote the formation of OxPLs in intestine, which *iii*) induce *Enpp2* and raise autotaxin levels in enterocytes, that *iv*) increases enterocyte lysophosphatidic acid (LPA) levels, that *v*) feedback to promote intestinal formation of ROS that maintain the high levels of intestinal OxPLs, that *vi*) decrease the levels of antimicrobial peptides and proteins in the intestine, that *vii*) lead to elevated levels of gut-derived LPS in plasma, that *viii*) enhance dyslipidemia, systemic inflammation, and atherosclerosis.

Smyth *et al.* (71) made the case for autotaxin-derived LPA playing a role in the development and complications of atherosclerosis. The studies presented here provide support for this hypothesis and show that targeting enterocyte *Enpp2* reduced WD-mediated increases in plasma LPS, dyslipidemia, and aortic atherosclerosis in *Ldlr*^*−/−*^ mice. Figure 14 presents a schematic diagram that combines our previous data (12, 13, 14, 16, 18, 22) with the data presented here to show potential mechanisms for the role of enterocyte *Enpp2* in these processes.

## Data availability

The data described in this article are all contained within the article. The reagents and transgenic animals can be shared upon request, by contacting either S. T. R. at sreddy@mednet.ucla.edu or A. M. F. at afogelman@mednet.ucla.edu.

## Supplemental data

This article contains supplemental data.

## Conflict of interest

A. M. F, M. N., and S. T. R. were principals in Bruin Pharma, and A. M. F. was an officer in Bruin Pharma during the course of some of these studies. All other authors declare that they have no conflicts of interest with the contents of this article.
