# Supplementary material for: Role of enterocyte Enpp2 and autotaxin in regulating lipopolysaccharide levels, systemic inflammation, and atherosclerosis
Source: J Lipid Res. 2023 Apr 12;64(5):100370. doi: 10.1016/j.jlr.2023.100370 (PMC10200992; doi:10.1016/j.jlr.2023.100370)
Supplement: Supplemental Figures S1–S9 and Tables S1–S3 [file mmc1.pdf]

SUPPLEMENTAL DATA – JLR-D-23-00066 R-1

Role of Enterocyte *Enpp2* and Autotaxin in Regulating Lipopolysaccharide Levels, Systemic Inflammation and Atherosclerosis

Arnab Chattopadhyay[1], Pallavi Mukherjee[1], Dawoud Sulaiman[1], Huan Wang[1], Victor Girjalva[1], Nasrin Dorreh[1], Jonathan P. Jacobs[2,4,5], Samuel Delk[1,6], Wouter H. Moolenaar[7], Mohamad Navab[1], Srinivasa T. Reddy[1,3,6*], and Alan M. Fogelman[1].

Department of Medicine, Division of Cardiology[1], The Vatche and Tamar Manoukian Division of Digestive Diseases[2], Department of Molecular and Medical Pharmacology[3], UCLA Microbiome Center[4], David Geffen School of Medicine at UCLA and the Division of Gastroenterology, Hepatology and Parenteral Nutrition, Veterans Administration Greater Los Angeles Healthcare System Los Angeles[5], Molecular Toxicology Interdepartmental Degree Program[6], Fielding School of Public Health, University of California, Los Angeles, CA 90095, USA; Division of Biochemistry, Netherlands Cancer Institute, Amsterdam, the Netherlands[7].

Supplemental Tables S1 – S3

Supplemental Figures S1 – S9

SUPPLEMENTAL TABLES

Supplemental Table S1. ELISA kits used in accordance with manufacturer's instructions.

Protein Assayed	Tissue Source	Tissue Dilution	Kit Source	Catalog #
APOA-I	Plasma	1:10000	Abcam	ab221440
ATOH1	Whole jejunum	1:100	Aviva Systems Biology	OKEH05889
DLL4	Whole jejunum	1:2	Abcam	Ab213860
GFI1	Whole jejunum	1:100	Abbexa	Abx525156
IL-6	Plasma	1:2	LS Bio	LS-F31413-1
IL22	Whole jejunum	1:2	Biolegend	436307
IL23	Whole jejunum	1:2	Biolegend	433704
IL36	Whole jejunum	1:10	Aviva Systems Biology	OKEH03002
LBP	Jejunum mucus<br><br>Plasma	No dilution<br><br>1:10	Enzo Life Sciences<br>Jackson Immuno Research	ALX-804-502-C100<br>315-035-0031
Lysozyme	Jejunum mucus	1:10	Lifeome	E91193Mu-1
Mucin2	Jejunum mucus	1:100	Lifeome	E15065-96
NOTCH2	Whole jejunum	1:2	Aviva Systems Biology	OKEH05163
Osteopontin	Jejunum mucus	No dilution	Abcam	Ab100734
SAA	Plasma	1:1000	Abcam	ab216059

Supplemental Table S2. Primers used for quantification of gene expression by RT-qPCR.

GENE	PRIMER
Alp1 (Forward)	TCCCAGTATGTTTGGAATCGTG
Alp1 (Reverse)	CGAACATCACAGCCTAGTCAG
ApoAI (Forward)	GTGGCTCTGGTCTTCCTGAC
ApoAI (Reverse)	ACGGTTGAACCCAGAGTGTC
Atoh1 (Forward)	AGCTTCCTCTGGGGGTTACT
Atoh1 (Reverse)	TTCTGTGCCATCATCGCTGT
Defb3 (Forward)	TCTGTTTGCATTTCTCCTGGTG
Defb3 (Reverse)	TAAACTTCCAACAGCTGGAGTGG
Defb4 (Forward)	TCTGTTTGCATTTCTCCTGGTG
Defb4 (Reverse)	TTTGCTAAAAGCTGCAGGTGG
Dll4 (Forward)	ACCTTT GGCAAT GTCTCC
Dll4 (Reverse)	GTTTCCTGGCGAAGTCTCTG
Enpp2 (Forward)	TCGAGGGCGAGAGAAGTTTA
Enpp2 (Reverse)	AAAAGAATGTCCCGGCTCTC
Gapdh (Forward)	TGTGTCCGTCGTGGATCTGA
Gapdh (Reverse)	CCTGCTTCACCACCTTCTTGAT
Gfi1 (Forward)	GAGCAACACAAGGCAGTG
Gfi1 (Reverse)	TCTTGCCACAGATCTTACAGTC
Il22 (Forward)	CATGCAGGAGGTGGTACCTT
Il22 (Reverse)	CAGACGCAAGCATTTCTCAG
Il23 (Forward)	AATAATGTGCCCCGTATCCAG T
Il23 (Reverse)	GCTCCCCTTTGAAGATGTCAG
Il36γ (Forward)	AGAGTAACCCCAGTCAGCGTG
Il36γ (Reverse)	AGGGTGGTGGTACAAATCCAA
Lbp (Forward)	GGAGGTCCACTGAAATGGTG
Lbp (Reverse)	TCGCCATCTCTGACTCTTCC
Lyz (Forward)	CTGGGACTCCTCCTGCTTTCTG
Lyz (Reverse)	CTTCGGTCTCCACGGTTGTAGT
Muc2 (Forward)	CCTTGCAGTCAAACTCAAAGT
Muc2 (Reverse)	AAGTTTGCCCCTGGCTATGAC
Notch1 (Forward)	CCCTTGCTCTGCCTAACG C
Notch1 (Reverse)	GGAGTCCTGGCATCGTTG G
Notch2 (Forward)	GGAATGGTGGC GAGTTGAT
Notch2 (Reverse)	TCGCCTCCACATTATTGACA
Reg3b (Forward)	GGCTTCATTCTTGTTGTCCTCCA
Reg3b (Reverse)	TCCACCTCCATTGGGTTCT
Reg3g (Forward)	AAGCTTCCTTCCTGTCCTCC
Reg3g (Reverse)	TCCACCTCTGTTGGGTTCAT
Sftpa1 (Forward)	AAAGGGGGCTTCCAGGGTTTCCAGC
Sftpa1 (Reverse	ATTCCTCGGGGCAGCAATGTGG
Spp1 (Forward)	CTTTCACTCCAATCGTCCCTAC
Spp1 (Reverse)	GCTCTCTTTGGAATGCTCAAGT

Supplemental Table S3. Statistical analysis of gene expression for the genes described in Figure 4 was performed as described in Methods.

Gene	***P* Value: Chow Cont. vs. Chow iKO**	***P* Value: Chow Cont. vs. WD Cont.**	***P* Value: Chow Cont. vs. WD iKO**	***P* Value: Chow iKO vs. WD Cont.**	***P* Value: Chow iKO vs. WD iKO**	***P* Value: WD Cont. vs. WD iKO**
Alp1	0.9126	0.0039	0.9210	0.0222	0.5760	0.0006
ApoA-I	0.5775	0.4979	0.9083	0.0490	0.2241	0.8771
Atoh1	0.0149	0.8381	0.3552	0.0015	0.4347	0.0171
Defb4	0.1738	0.0707	0.9995	0.0019	0.0550	0.0560
Dll4	0.0001	0.0458	0.6807	<0.0001	<0.0001	0.3484
Gfi1	0.1746	0.0250	0.8105	0.0005	0.6281	0.0135
Il22	0.8209	0.0029	0.7849	0.0002	0.9999	0.0002
Il23	0.7273	0.7458	0.0447	0.0479	0.7273	0.0193
Il36g	0.5562	0.3460	0.0081	0.0237	0.1717	<0.0001
Lyz	0.5501	0.3517	0.9960	0.0238	0.4168	0.4773
Muc2	0.9931	0.0113	0.4830	0.0280	0.3335	0.6134
Notch1	0.8794	0.0002	0.3050	0.0021	0.7356	0.0315
Notch2	0.7200	0.0212	0.7804	0.0012	0.2074	0.0423
Reg3b	0.7920	0.0146	0.0643	0.0012	0.0065	0.9262
Reg3g	0.0078	0.4478	0.8726	0.0001	0.0009	0.8793
Sftpa1	NS	NS	NS	NS	NS	NS
Defb3	NS	NS	NS	NS	NS	NS
Spp1	>0.9999	<0.0001	0.9967	<0.0001	0.9986	<0.0001
Lbp	0.3880	0.0034	0.9912	0.0389	0.2654	0.0020

SUPPLEMENTAL FIGURES

Supplemental Figure S1.

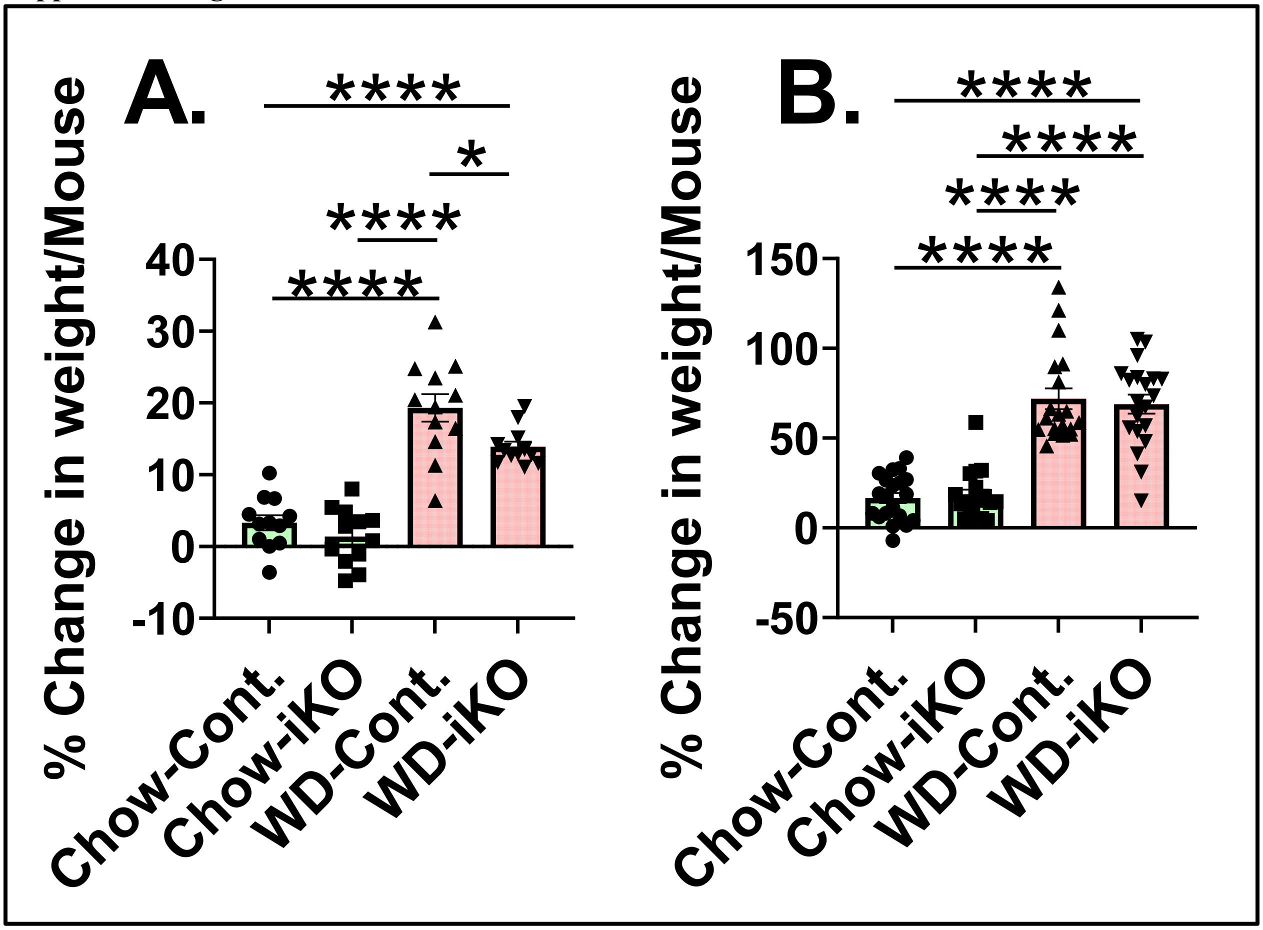

Supplemental Figure S1. Weight change by genotype and diet. (**A**) Female *Enpp2*$^{fl/fl}$/*Ldlr*$^{-/-}$ (Cont.) mice 3.3 ± 0.3 months of age (n = 12 mice per group) and female *Enpp2*$^{fl/fl}$/*Ldlr*$^{-/-}$/*VilCre* (iKO) mice 3.4 ± 0.3 months of age (n = 12 mice per group) were fed either the chow diet (Chow) or the Western diet (WD). After two weeks, the percent change in weight was determined for each mouse. (**B**) Female Cont. mice 2.6 ± 0.1 months of age (n = 19 to 20 mice per group) and female iKO mice 2.8 ± 0.1 months of age (n = 18 to 20 mice per group) were fed either Chow or WD. After twenty weeks, the percent change in weight was determined for each mouse * $P < 0.05$; **** $P < 0.0001$.

Supplemental Figure S2.

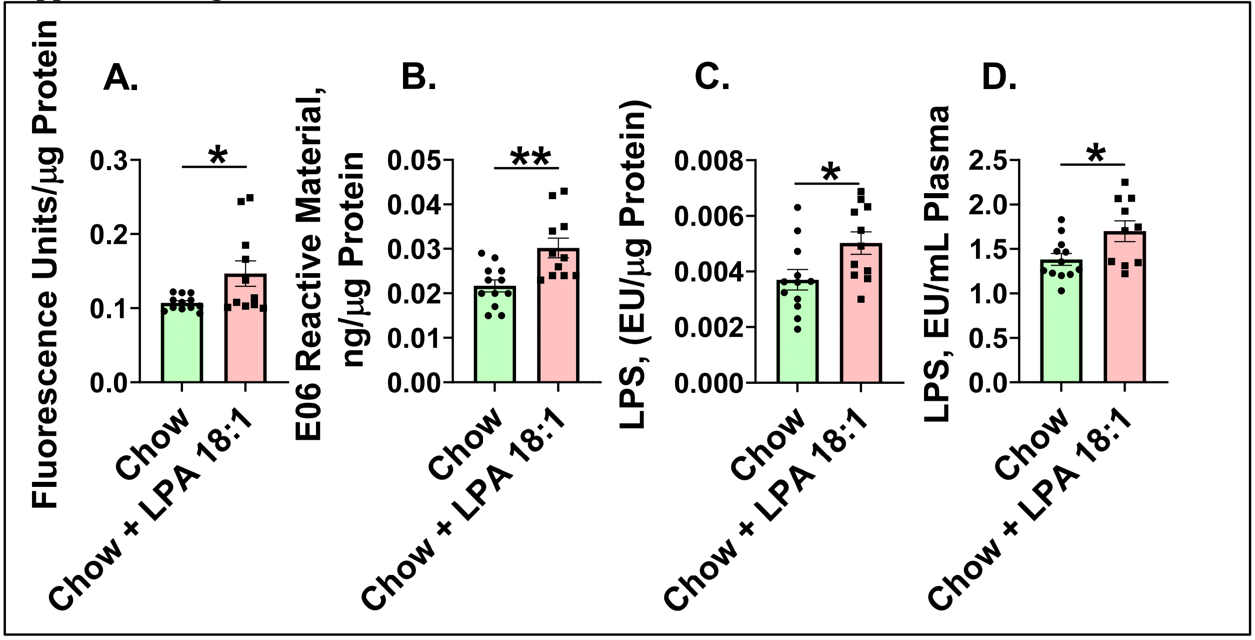

Supplemental Figure S2. Adding lysophosphatidic acid (LPA) 18:1 to standard mouse chow resulted in changes in jejunum mucus qualitatively similar to those seen on feeding a Western diet (WD) to *Ldlr*$^{-/-}$ mice. Male *Ldlr*$^{-/-}$ mice 3 months of age (n = 12 per group) were fed chow or chow supplemented with 1 µg per gram chow of LPA 18:1. After 3 weeks, jejunum mucus and plasma were collected, and levels of reactive oxygen species (**A**), oxidized phospholipids (**B**), and bacterial lipopolysaccharide (LPS) (**C**) in jejunum mucus or LPS in plasma (**D**) were determined as described in Methods. **P* < 0.05; ***P* < 0.01.

Supplemental Figure S3.

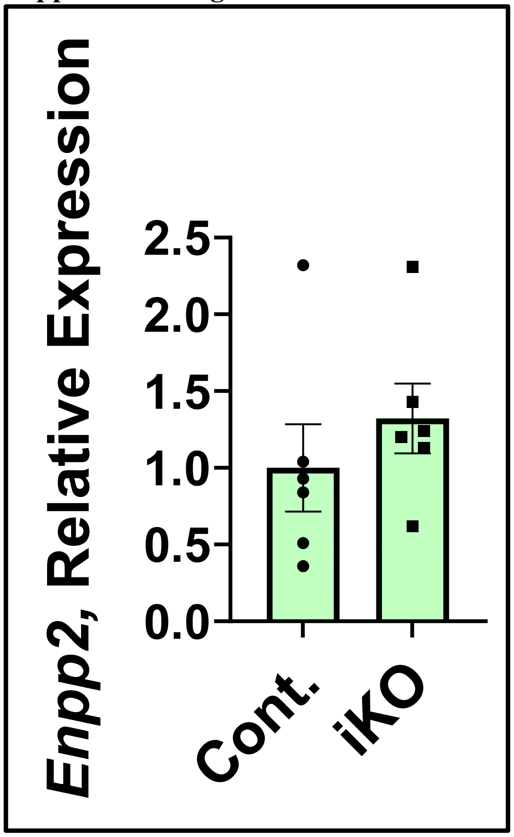

Supplemental Figure S3. Hepatic expression of *Enpp2* was not altered in *Enpp2*$^{fl/fl}$/*Ldlr*$^{-/-}$/*VilCre* (iKO) mice. *Enpp2* gene expression in the livers of female *Enpp2*$^{fl/fl}$/*Ldlr*$^{-/-}$ (Cont.) mice 3.2 ± 0.6 months of age (n = 6 mice per group) and in female iKO mice 4.2 ± 0.5 months of age (n = 6 mice per group) was determined by RT-qPCR as described in Methods.

Supplemental Figure S4.

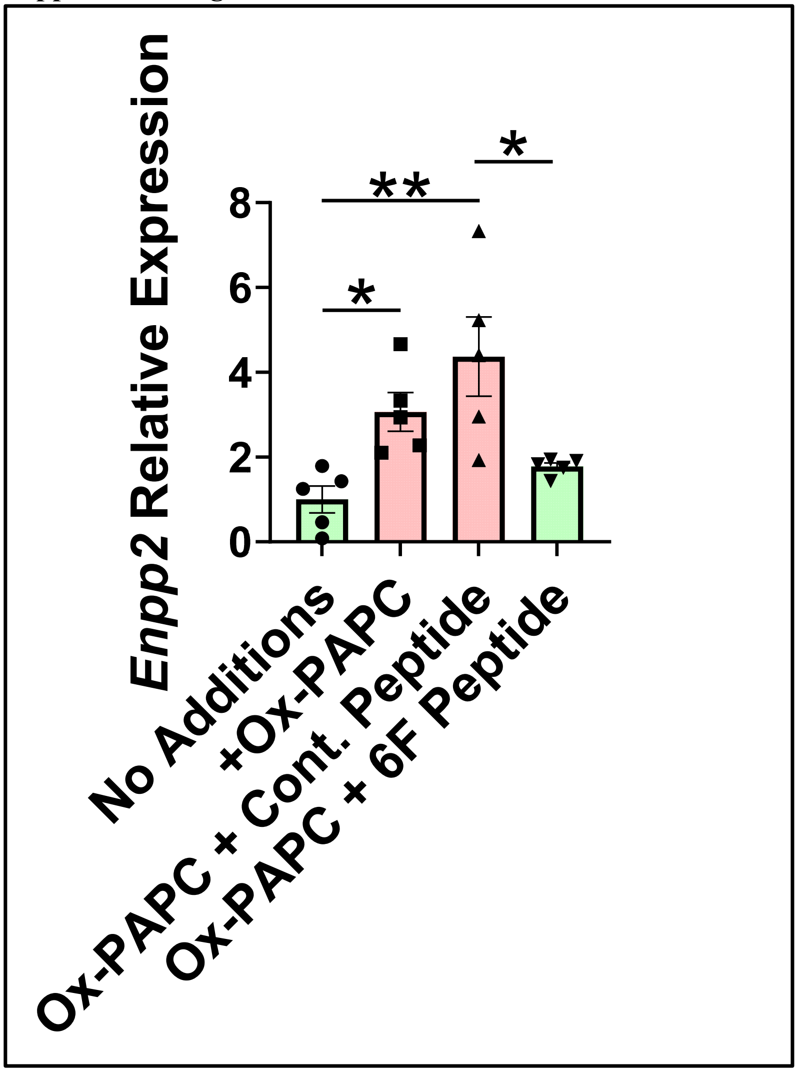

Supplemental Figure S4. Adding oxidized phospholipids *ex vivo* to jejunum from mice on a chow diet reproduces *in vivo* gene expression for *Enpp2* seen in jejunum from *Ldlr*$^{-/-}$ mice fed a Western diet. Jejunum segments from male *Ldlr*$^{-/-}$ mice on a chow diet 3 months of age (n = 5 per group) were incubated with no additions, or with 25 µg of Ox-PAPC without or with 25 µg of a control (Cont.) peptide or 25 µg of 6F peptide as described in Methods. After 4 h of incubation gene expression for *Enpp2* was determined in the jejunum segments by RT-qPCR as described in Methods. * $P < 0.05$; ** $P < 0.01$.

Supplemental Figure S5.

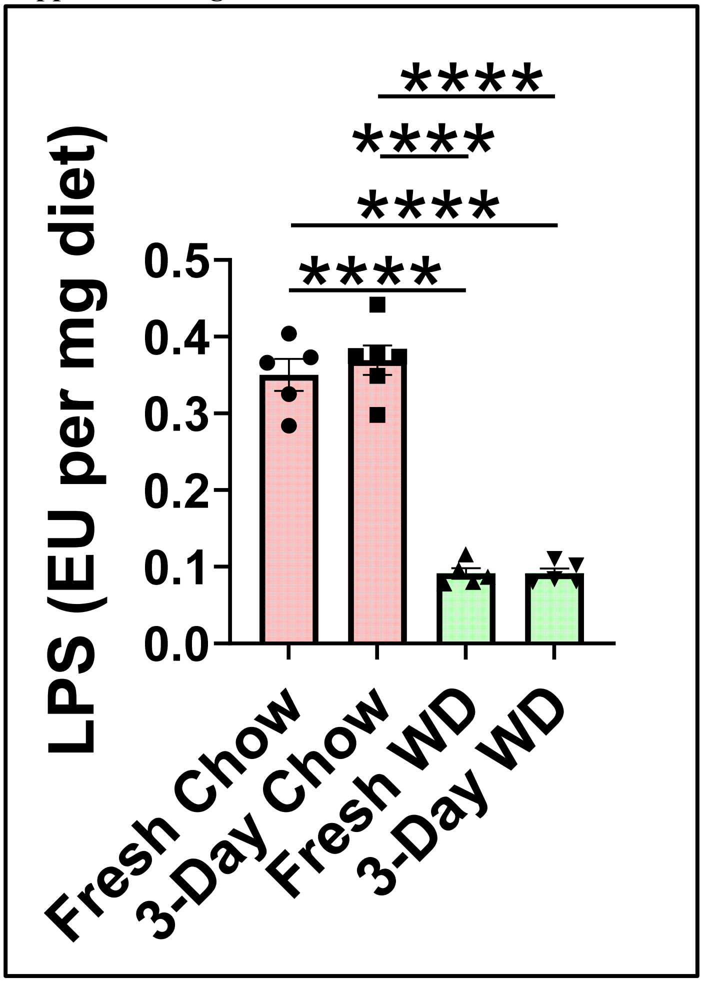

Supplemental Figure S5. Bacterial lipopolysaccharide (LPS) levels in mouse chow (Chow) were higher than in a Western diet (WD). Fresh diet pellets (normal mouse chow or WD) or pellets that were left in the feeding rack of the mouse cages for 3 days (3-Day) were ground to powder in a blender, and 100 to 150 mg of the resulting powder was sonicated in 500 µL of endotoxin-free water, and centrifuged in a Beckman Coulter Microfuge 18 at 14,000 RPM for 10 minutes. The supernatant was centrifuged through a 22-micron filter in the same centrifuge at 14,000 RPM, the flow through was diluted 1:25 with endotoxin-free water, and LPS was measured using a Pierce Chromogenic Endotoxin Quant Kit (Thermo Scientific, catalog #A39553) according to the manufacturer's instructions. The EU values were normalized to the weights of the initial samples. ****$P < 0.0001$.

Supplemental Figure S6.

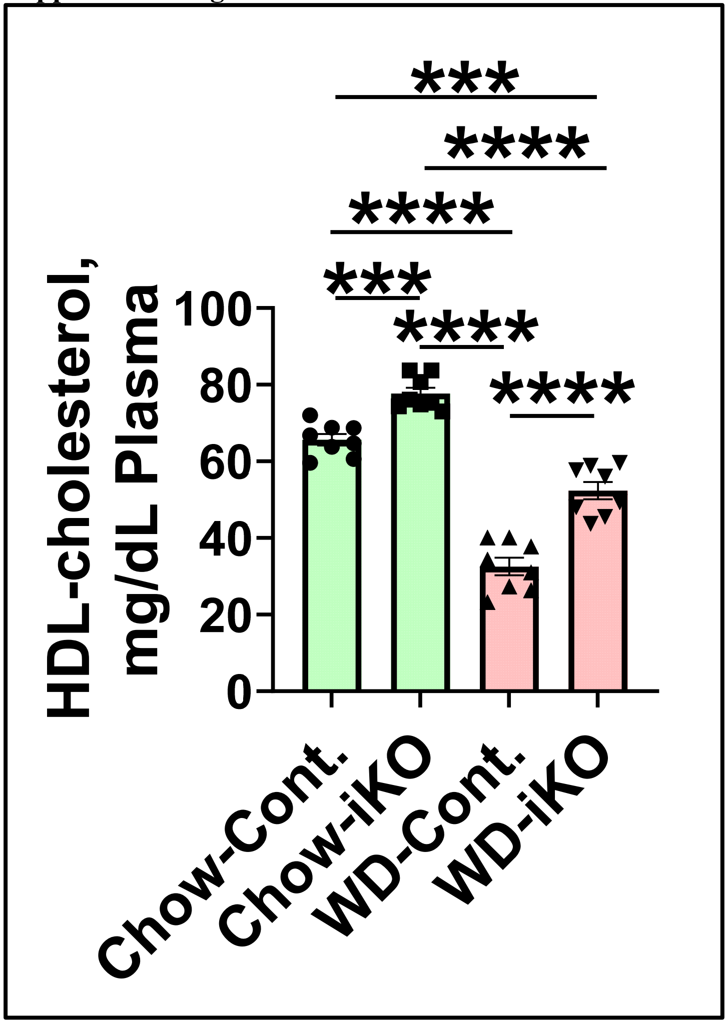

Supplemental Figure S6. Plasma HDL-cholesterol levels on the chow diet (Chow) or the Western diet (WD). Male *Enpp2*$^{fl/fl}$/*Ldlr*$^{-/-}$ (Cont.) mice 7.4 ± 0.3 months of age (n = 8 per group), or male *Enpp2*$^{fl/fl}$/*Ldlr*$^{-/-}$/*VilCre* (iKO) mice 7.3 ± 0.3 months of age (n = 8 per group) were fed Chow or WD. After two weeks, plasma HDL-cholesterol levels were determined as described in Methods. ***$P < 0.001$; ****$P < 0.0001$.

Supplemental Figure S7.

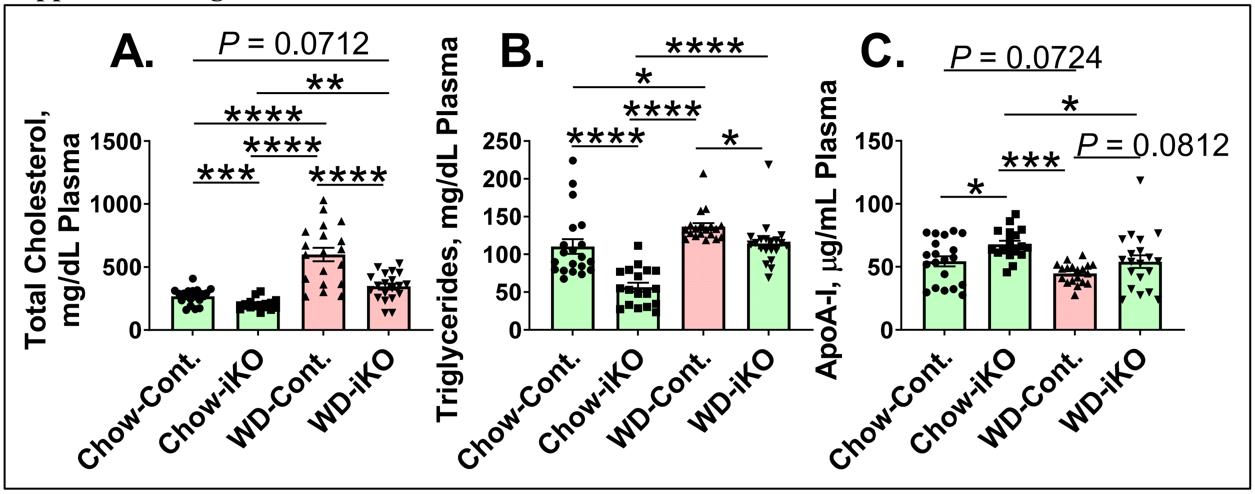

Supplemental Figure S7. Plasma total cholesterol, triglycerides and apoA-I after five months on the chow diet (Chow) or the Western diet (WD). Female *Enpp2*$^{fl/fl}$/*Ldlr*$^{-/-}$ (Cont.) mice 2.6 ± 0.1 months of age (n = 19 to 20 mice per group) and *Enpp2*$^{fl/fl}$/*Ldlr*$^{-/-}$/*VilCre* (iKO) mice 2.8 ± 0.1 months of age (18 to 20 mice per group) were fed either Chow or WD. After five months, plasma levels of total cholesterol (**A**), triglycerides (**B**) and apoA-I (**C**) were determined as described in Methods. * $P < 0.05$; ** $P < 0.01$; *** $P < 0.001$; **** $P < 0.0001$.

Supplemental Figure S8.

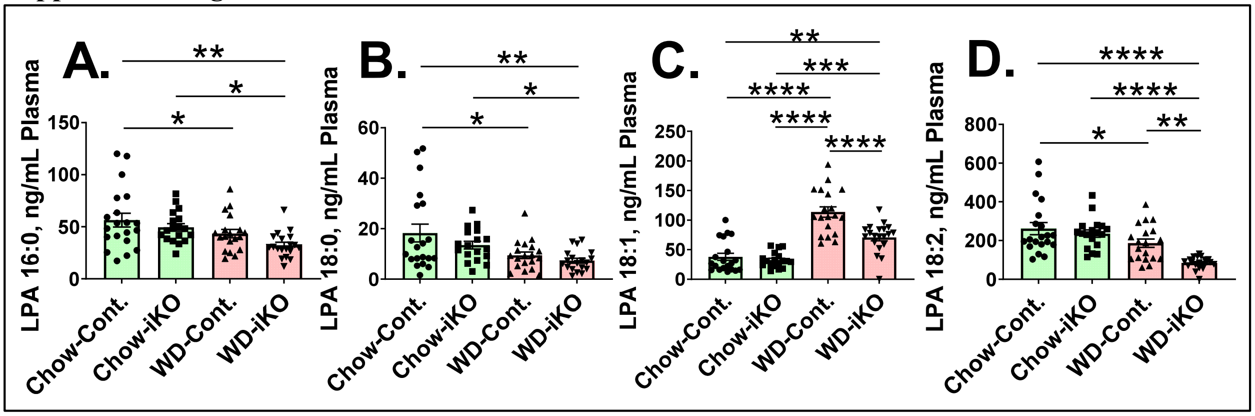

Supplemental Figure S8. Plasma LPA levels after 5 months on the chow diet (Chow) or the Western diet (WD). The mice described in Supplemental Figure S7 [Female *Enpp2*$^{fl/fl}$/*Ldlr*$^{-/-}$ (Cont.) mice 2.6 ± 0.1 months of age (n = 19 to 20 mice per group) and *Enpp2*$^{fl/fl}$/*Ldlr*$^{-/-}$/*VilCre* (iKO) mice 2.8 ± 0.1 months of age (n = 18 to 20 mice per group)] were fed either Chow or WD. After five months the levels of LPA 16:0 (**A**), LPA 18:0 (**B**), LPA 18:1(**C**) and LPA 18:2 (**D**) were determined in plasma as described in Methods. * $P < 0.05$; ** $P < 0.01$; *** $P < 0.001$; **** $P < 0.0001$.

Supplemental Figure S9.

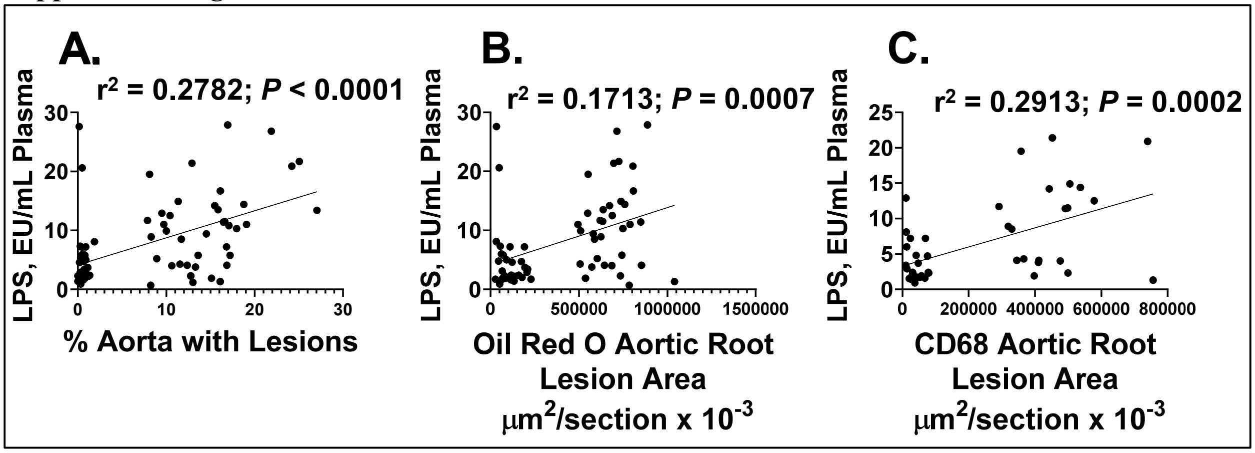

Supplemental Figure S9. Plasma LPS levels correlate with aortic lesions. Linear regression was performed for the LPS data from each mouse in Figure 12B versus the lesion data from the same mouse in Figures 13A (**A**), 13B (**B**), and 13C (**C**).
